# Unfolded Protein Response Inhibition Reduces Middle East Respiratory Syndrome Coronavirus-Induced Acute Lung Injury

**DOI:** 10.1128/mBio.01572-21

**Published:** 2021-08-10

**Authors:** Amy C. Sims, Hugh D. Mitchell, Lisa E. Gralinski, Jennifer E. Kyle, Kristin E. Burnum-Johnson, Mariam Lam, M. Leslie Fulcher, Ande West, Richard D. Smith, Scott H. Randell, Thomas O. Metz, Timothy P. Sheahan, Katrina M. Waters, Ralph S. Baric

**Affiliations:** a Department of Epidemiology, University of North Carolina at Chapel Hill, Chapel Hill, North Carolina, USA; b Biological Sciences Division, Pacific Northwest National Laboratories, Richland, Washington, USA; c Marsico Lung Institute Tissue Procurement and Cell Culture Core, University of North Carolina at Chapel Hill, Chapel Hill, North Carolina, USA; d Department of Cell Biology and Physiology, University of North Carolina at Chapel Hill, Chapel Hill, North Carolina, USA; Columbia University Medical College

**Keywords:** MERS-CoV, acute lung injury, primary human lung cells, microvascular endothelial cells, fibroblasts, apoptosis, unfolded protein response

## Abstract

Tissue- and cell-specific expression patterns are highly variable within and across individuals, leading to altered host responses after acute virus infection. Unraveling key tissue-specific response patterns provides novel opportunities for defining fundamental mechanisms of virus-host interaction in disease and the identification of critical tissue-specific networks for disease intervention in the lung. Currently, there are no approved therapeutics for Middle East respiratory syndrome coronavirus (MERS-CoV) patients, and little is understood about how lung cell types contribute to disease outcomes. MERS-CoV replicates equivalently in primary human lung microvascular endothelial cells (MVE) and fibroblasts (FB) and to equivalent peak titers but with slower replication kinetics in human airway epithelial cell cultures (HAE). However, only infected MVE demonstrate observable virus-induced cytopathic effect. To explore mechanisms leading to reduced MVE viability, donor-matched human lung MVE, HAE, and FB were infected, and their transcriptomes, proteomes, and lipidomes were monitored over time. Validated functional enrichment analysis demonstrated that MERS-CoV-infected MVE were dying via an unfolded protein response (UPR)-mediated apoptosis. Pharmacologic manipulation of the UPR in MERS-CoV-infected primary lung cells reduced viral titers and in male mice improved respiratory function with accompanying reductions in weight loss, pathological signatures of acute lung injury, and times to recovery. Systems biology analysis and validation studies of global kinetic transcript, protein, and lipid data sets confirmed that inhibition of host stress pathways that are differentially regulated following MERS-CoV infection of different tissue types can alleviate symptom progression to end-stage lung disease commonly seen following emerging coronavirus outbreaks.

## INTRODUCTION

Coronaviruses (CoV) are important emerging pathogens associated with severe disease outcomes in humans and animals causing significant global morbidity and mortality. In 2003, severe acute respiratory coronavirus (SARS-CoV) emerged from closely related bat CoV strains, spread to 26 nations, and caused approximately 8,000 human cases and 800 deaths worldwide (1, 2). In 2012, Middle East respiratory syndrome CoV (MERS-CoV) emerged, causing approximately 2,500 cases with an ∼35% mortality rate in 27 countries, and the outbreak is still ongoing (3). In 2020, the causative agent of coronavirus infectious disease 2019 (COVID-19) was identified as severe acute respiratory syndrome coronavirus 2 (SARS-CoV2), and, to date, >120 million cases have been confirmed, with a morality rate of ∼3% in >200 countries/territories, with ongoing virus transmission resulting in significant economic losses and global public health concerns (WHO COVID-19 website). SARS-CoV2 and SARS-CoV disease severity are strongly influenced by aging and other comorbidities (e.g., diabetes and obesity), and mortality rates approach 15% (>60 years) or exceed 50%, respectively (4). These data underscore the highly pathogenic potential of emerging CoVs and reinforce the critical need for comprehensive experimental approaches that define the viral and host pathogenic programs that could be targeted by countermeasures to reverse severe disease outcomes.

Acute respiratory distress syndrome (ARDS) is a severe end-stage lung disease characterized by rapid-onset respiratory failure resulting from diffuse alveolar damage, hyaline membrane formation, vascular leakage into the airways, and pulmonary fibrosis (5–8). ARDS is a complex syndrome with multiple etiologies, complicating diagnosis and treatment. While not the most prevalent cause (9), severe avian influenza (10) (H5N1) and SARS-CoV, SARS-CoV2, and MERS-CoV (2, 11–14) respiratory infections can progress from acute lung injury (ALI; a less severe form of ARDS) to ARDS. Dissecting the mechanisms of MERS-CoV-induced ALI and ARDS has been hampered by human genetic variation, variable comorbidities, and limited human sample availability (11, 15). Evidence of programmed cell death has been detected in models of coronavirus pathogenesis and in patient autopsy tissues (4, 16–23). Because MERS-CoV replicates efficiently in multiple cell types essential for lung physiology and organ homeostasis (e.g., lung fibroblasts [FB] and epithelial and endothelial cells) (24), primary cell culture systems provide a platform to better understand virus and virus-mediated tissue-specific host interactions that may drive severe lung pathology.

Here, we report a kinetic multiomics analysis of matched MERS-CoV-infected primary human lung FB, conducting airway epithelial cells (HAE), and microvascular endothelial cells (MVE) from three different human donors. The cell types were chosen to represent the most diverse primary cell types permissive for MERS-CoV infection that also had established culture systems amenable to our study design, providing a model platform to investigate differential tissue-specific host response patterns prior to infection. While MERS-CoV replication kinetics were similar among FB, HAE, and MVE, the host responses, as determined by multiomic transcriptomic, proteomic, and lipidomic analyses, differed significantly by cell type and suggested that MVE cells were dying via apoptotic mechanisms initiated by the unfolded protein response (UPR), which helps monitor and maintain homeostasis in the endoplasmic reticulum (ER) following stress response activation. Pharmacologic manipulation of the UPR demonstrated that active/dimerized PERK was critical for viral replication *in vitro*. More importantly, mice infected with MERS-CoV and treated with a PERK-specific inhibitor demonstrated reduced weight loss, pneumonia, and ALI, leading to improved respiratory function and time to recovery. These data not only articulate an important role for the epithelial-endothelial cell barrier in MERS-CoV pathogenesis and ALI but also reveal the importance of interrogating tissue-specific response patterns for identifying novel host-targeted therapeutic options for the treatment of emerging coronavirus and other viral infections in the lung.

## RESULTS

### Cross-donor consensus transcriptomic, proteomic, and lipidomic responses following MERS-CoV infection.

**FIG 1:**
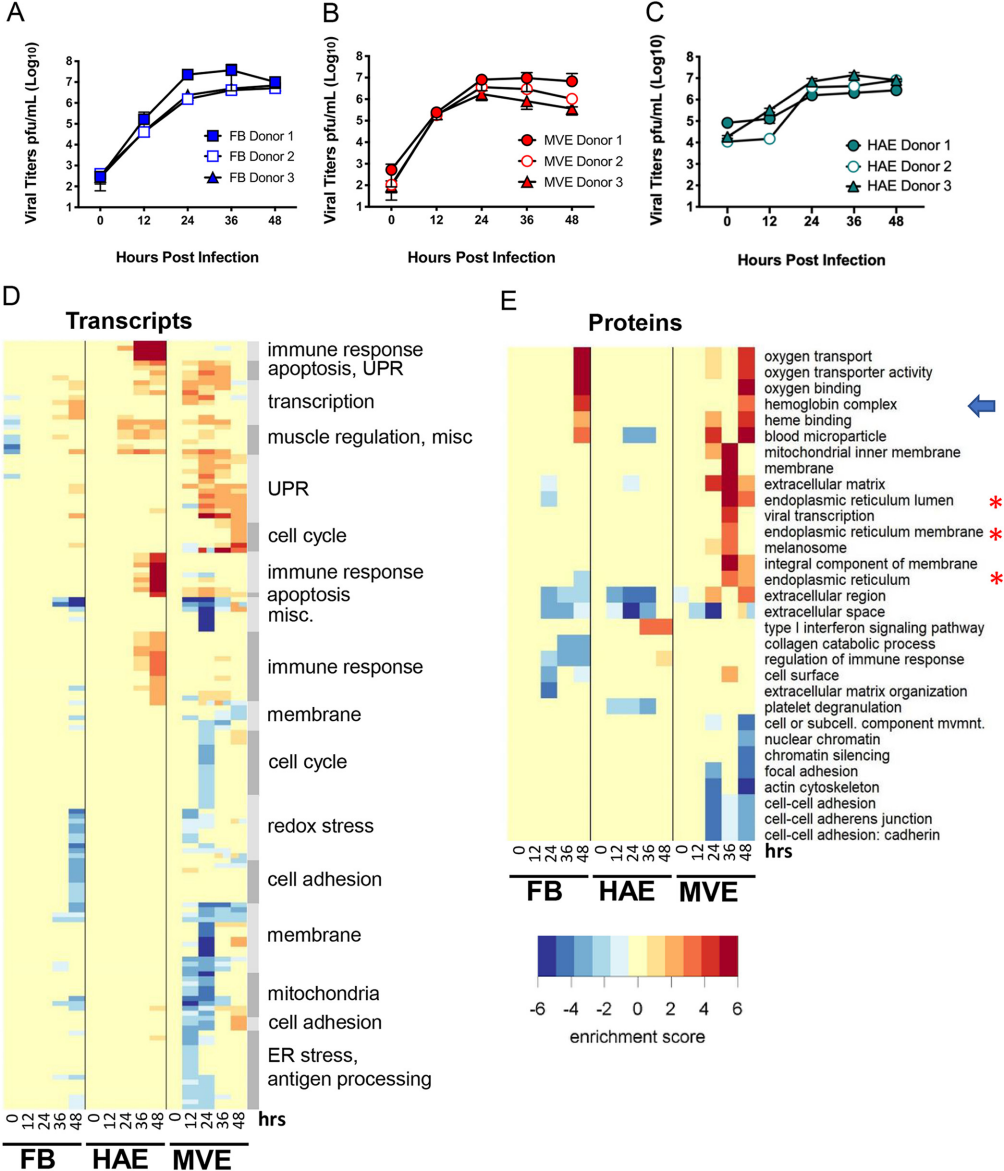
Transcriptomics and proteomics suggest activation of unfolded protein response and apoptotic pathways in MERS-CoV-infected primary human lung microvascular endothelial cells but not in infected airway epithelial cell cultures or fibroblasts. (A to C) Viral replication. Donor-matched microvascular endothelial cells (MVE), human airway epithelial cell cultures (HAE), and fibroblasts (FB) were infected with wild-type MERS-CoV (MOI of 5), and supernatants were collected at the indicated times and viral titers determined by plaque assay. Results are shown as plaque forming units (PFU) per ml over time. Each data point represents averaged data from supernatant collected from 10 different wells (5 wells harvested for RNA and 5 wells fractionated for proteins and lipids). The graphs in panels A, B, and C show levels of replication detected for all three tissue donors in all three cell types. Error bars indicate standard deviations from the means. (D) Functional enrichment was performed on transcriptomic data from all three donor samples in both cell types, and results were only retained that were present in all three donors, keeping only the least significant score. In this way, a true consensus response is represented by all indicated functions. (E) Functional enrichment was performed on proteomic data from all three donor samples in both cell types, and results were only retained that were present in all three donors, as in panel D. The blue arrow highlights proteins in the hemoglobin complex, and red asterisks highlight apoptotic proteins from the endoplasmic reticulum.

Identifying MERS-CoV pathogenic mechanisms requires an understanding of critical virus-host interactions in infected primary human lung cells that provide the vital structural and physiological requirements for lung function. As clinical samples for MERS-CoV are scarce, using donor-matched primary human lung cells from previously healthy donors provides a novel opportunity to elucidate tissue-specific changes that may explain disease phenotypes. To characterize MERS-CoV infection in primary human lung airway epithelial cell cultures (HAE), primary human lung microvascular endothelial cells (MVE), and primary human lung fibroblasts (FB), we infected cells from three matched human donors at a multiplicity of infection (MOI) of 5 and conducted virologic and multiomic (transcriptomic, proteomic, and lipidomic) analyses at 0, 12, 24, 36, and 48 h postinfection (hpi). Interestingly, viral growth kinetics and peak titers (∼10^7^ PFU/ml) were nearly identical regardless of donor background (Fig. 1A to C), with a slight expected delay in viral replication kinetics in HAE cultures. A previous study reported distinct replication levels between donors when infected with MERS-CoV, but this difference may be explained by the lower MOI used in the earlier study (24). To gain insight into the host pathways that were uniquely or commonly differentially regulated by MERS-CoV infection of HAE, FB, and MVE, functional enrichment was performed on donor-matched kinetic transcriptomic and proteomic data across all three cell types, using an approach where only the least significant response of the three donors is preserved for each ontology term. This conservative enrichment strategy takes full advantage of our unique human donor data set and ensures that only responses that are most likely to be conserved across the human population are displayed. We found distinct differences in the modulation of the transcriptome and proteome among infected HAE, FB, and MVE (Fig. 1D and E), revealing pathways related to apoptosis (transcription; Fig. 1D) and the unfolded protein response (UPR; transcripts and proteins; Fig. 1D and E, asterisks), were significantly enriched in MERS-CoV-infected MVE at multiple time points but not observed in similarly infected HAE or FB. Interestingly, immune-related responses were detected only in HAE transcriptomics, suggesting that this is the only cell type of the three that programs an innate immune response and suggests a marked vulnerability of lung FB and MVE to infection. Susceptibility of MVE and FB to MERS-CoV infection may also contribute to the higher mortality rates for MERS-CoV patients compared to SARS-CoV- and SARS-CoV2-infected individuals, since the latter viruses do not infect FB and MVE in infection models (24, 25). We also observed increased hemoglobin protein expression levels in FB and MVE at 48 h postinfection (Fig. 1E, arrow), which has been demonstrated to regulate endothelial and epithelial cell communication as well as to regulate the durability of vascular walls (26–28). This increase was not detected in infected HAE.

**FIG 2:**
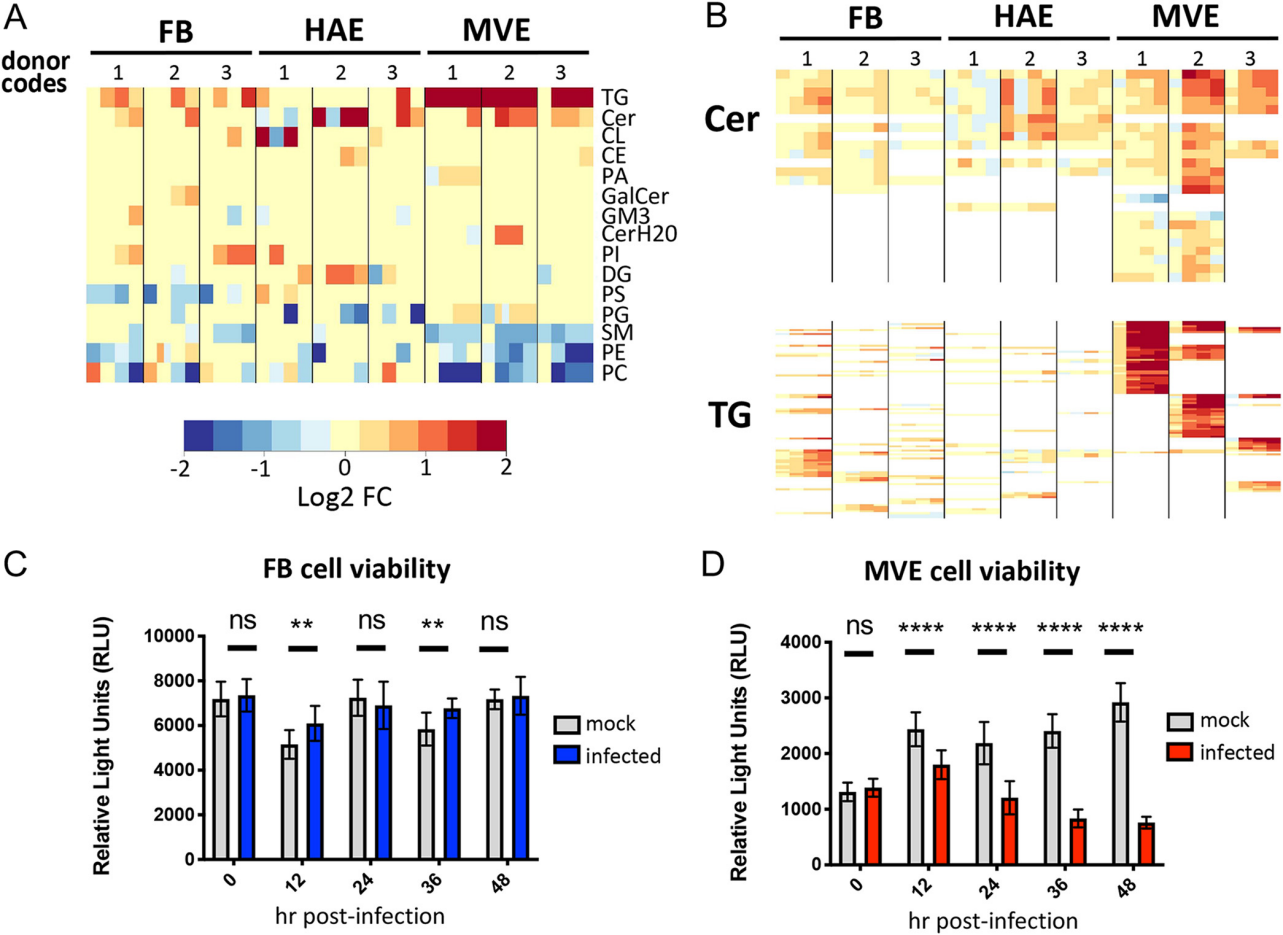
Lipidomics and cell viability assessment support activation of apoptotic pathways in MERS-CoV-infected microvascular endothelial cells but not fibroblasts. (A) Enrichment analysis using lipid classes as enrichment sets. TG, triglycerides; Cer, ceramide; CL, cardiolipins; CE, cholesterol esters; PA, phosphatidic acid; GalCer, galactosylceramide; GM3, GM3 ganglioside; PI, phosphatidylinositol; DG, diglycerides; PS, phosphatidylserine; PG, phosphatidylglycerol; SM, sphingomyelin; PE, phosphatidylethanolamine; PC, phosphatidylcholine. (B) Abundance of individual lipid species, whose fold change compared to mock-infected samples was at a *P* value of 0.001 or below in at least one condition and was a member of one of the indicated lipid classes (triglycerides or ceramides). (C and D) Cell viability following MERS-CoV infection. Donor-matched MVE and FB were infected with wild-type MERS-CoV and assessed for cell viability using the CellTiter-Glo kit according to the manufacturer’s instructions (Promega) at the indicated times postinfection. Relative light units were graphed over time, and error bars indicate standard deviations from the means. Statistical analysis was performed in GraphPad and determined by Mann-Whitney U test. Gray bars, mock-infected cells; red bars, MERS-CoV-infected MVE; blue bars, MERS-CoV-infected FB; ****, *P* < 0.0001; **, *P* < 0.002; ns, not statistically significant.

We also sought to determine if MERS-CoV infection alters the lipidome in HAE, FB, and/or MVE. We classified lipids into broad categories following enrichment analysis, which revealed differential expression of ceramides and triglycerides in HAE, FB, and MVE (Fig. 2A). When analyzed at a per-lipid-species level, we found dramatic and significant differences in specific species of ceramides (Fig. 2B, upper) and triglycerides (Fig. 2B, lower) in MERS-CoV-infected MVE, both of which are involved in activating apoptotic processes (29). Given their apparent immune vulnerability and the likely contrast in apoptotic/UPR pathway response between FB and MVE, we focused our further studies on these two cell types.

### MERS-CoV induces cytopathic effect in primary human lung endothelial cells but not fibroblasts.

To determine how the differences observed from omics studies affected cell viability when the cell types were compared, we used microscopy to determine total numbers of mock-infected and MERS-CoV-infected MVE and FB per field at each time point (see Fig. S1 in the supplemental material). For both mock-infected and infected FB, the total numbers of cells increased through 36 h postinfection with surprisingly little difference between mock-infected or infected cell counts at any time postinfection. In contrast, while the number of mock-infected MVE was steady over the course of the infection, numbers of infected MVE significantly declined over time (Fig. S1). Since the number of cells per field in mock-infected MVE cultures did not diminish over time, virus-induced cytopathic effect (CPE) was likely mediating the loss of cells in MERS-CoV-infected MVE (Fig. S1). To quantitate CPE in MERS-CoV-infected MVE and FB over time, we performed a cell viability assay (CellTiter-Glo), which estimates the number of metabolically active viable cells by measuring the levels of ATP in each well. Unlike FB, which had similar cell viability estimates in mock-infected and infected cells, infected MVE viability decreased significantly over time compared to mock (Fig. 2C and D, Fig. S2D to F), suggesting an infection-induced loss in cell viability. Together, these data demonstrate that MERS-CoV infections in both primary MVE and FB are similarly productive yet differentially regulate distinct host pathways leading to significantly reduced MVE, but not FB, cell viability over time.

10.1128/mBio.01572-21.1 FIG S1 Total cell counts of MERS-CoV-infected primary human lung MVE and FB. Donor-matched MVE and FB were mock-infected or infected with MERS-CoV expressing the red fluorescent protein (MERS-RFP), nuclei were stained with Hoechst stain, and images were captured at the indicated times postinfection. Total numbers of cells (assessed as numbers of nuclei per field) and total numbers of infected (contained red fluorescence) cells were assessed for at least three fields per condition using Fiji/ImageJ’s counting tool and plotted. Open blue bars, mock-infected FB; open red bars, mock-infected MVE; filled blue bars, MERS-RFP infected FB; filled red bars, MERS-CoV RFP infected MVE. Download FIG S1, PDF file, 0.05 MB. Copyright © 2021 Sims et al. 2021 Sims et al. https://creativecommons.org/licenses/by/4.0/ This content is distributed under the terms of the Creative Commons Attribution 4.0 International license.

10.1128/mBio.01572-21.2 FIG S2 MERS-CoV reduces cell viability for all three donors in MVE but not FB. Donor-matched FB (A to C) and MVE (D to F) cells were infected with wild-type MERS-CoV and assessed for cell viability using the CellTiter-Glo kit according to manufacturer’s instructions (Promega) at the indicated times post infection. Relative light units (RLU) were graphed over time, and error bars indicate standard deviation from the mean. Statistical analysis was performed in GraphPad determined by Mann-Whitney U test. Gray bars, mock-infected cells; red bars, MERS-CoV-infected MVE; blue bars, MERS-CoV-infected FB. ****, *P* < 0.0001; **, *P* <0.002; ns, not statistically significant. Download FIG S2, PDF file, 0.3 MB. Copyright © 2021 Sims et al. 2021 Sims et al. https://creativecommons.org/licenses/by/4.0/ This content is distributed under the terms of the Creative Commons Attribution 4.0 International license.

We then investigated if mothers against decapentaplegic homolog 7 (SMAD7) and/or fibroblast growth factor 2 (FGF2) transcripts were differentially expressed in MERS-CoV-infected MVE or FB, as previous studies indicated that upregulated expression of SMAD7 and FGF2 in MERS-CoV-infected immortalized kidney and lung epithelial cells led to cellular apoptosis (30). Neither SMAD7 nor FGF2 expression levels were distinct following infection and did not appear to explain the differences in cytopathic effect seen in the current studies (Fig. S3).

10.1128/mBio.01572-21.3 FIG S3 Neither SMAD7 nor FGF2 expression contributes to MERS-CoV-induced cytopathic effect in primary human lung cells. (A and B) Graphs show transcript expression levels for SMAD7 and FGF2 in MERS-CoV-infected MVE and FB over the 48-h time course (derived from microarray analysis and from the same datasets used in Fig. 1). Values graphed are fold change (log_2_), and error bars are standard deviations from the mean. SMAD7, mothers against decapentaplegic homolog 7; FGF2, fibroblast growth factor 2. Download FIG S3, PDF file, 0.06 MB. Copyright © 2021 Sims et al. 2021 Sims et al. https://creativecommons.org/licenses/by/4.0/ This content is distributed under the terms of the Creative Commons Attribution 4.0 International license.

### MERS-CoV activates caspases 3 and 7 in infected MVE.

**FIG 3:**
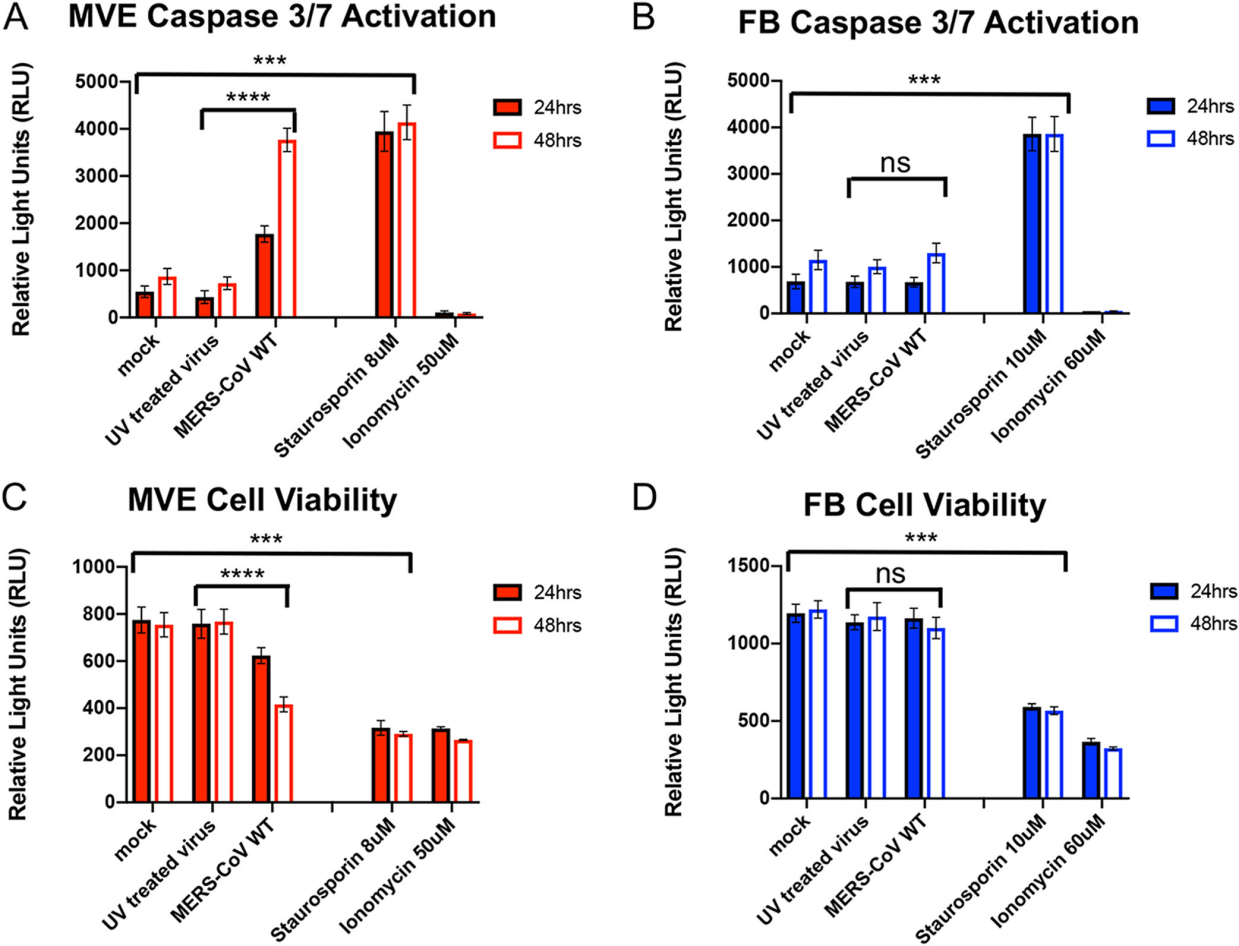
Caspases 3/7 are activated following MERS-CoV infection of primary human lung MVE but not primary lung FB. (A to D) Donor-matched MVE and FB were plated and mock infected or infected with UV-inactivated or wild-type MERS-CoV (MOI of 5), and, at 24 and 48 h postinfection, caspase 3/7 activation or cell viability was determined according to the manufacturer’s instructions using the Apotox Triplex kit (each well was measured for both parameters). Uninfected cells were treated with staurosporine (8 μM MVE/10 μM FB) to determine the maximal amount of caspase 3/7 activation. Uninfected cells were treated with ionomycin (50 μM MVE/60 μM FB) to demonstrate loss of cell viability via a nonapoptotic pathway. Results are graphed as relative light units, and error bars indicate standard deviations from the means. Statistical analysis was performed in GraphPad and determined by Mann-Whitney U test. (A and B) Results from caspase 3/7 activation. (C and D) Results from cell viability assay. Solid red bars, MVE 24-h samples; open red bars, MVE 48-h samples; solid blue bars, FB 24-h samples; open blue bars, FB 48-h samples. ****, *P* < 0.0001; ***, *P* < 0.002; ns, not statistically significant.

To validate the complementary multiomics observations suggesting that MERS-CoV infection induces apoptosis in MVE, we simultaneously measured death effector caspases 3 and 7 and cell viability in mock- or MERS-CoV-infected FB and MVE using the Promega Apotox Triplex kit. We included staurosporine (induces caspase 3/7 activation) or ionomycin (induces necrotic cell death with no caspase activation) treatment as controls. In addition, we added UV-inactivated MERS-CoV virions in order to control for effects on cultures independent of virus replication (i.e., entry, uncoating, etc.). Uninfected MVE and FB treated with staurosporine significantly induced caspase 3/7 activation compared to mock and cells treated with UV-inactivated MERS-CoV (Fig. 3A and B). As expected, ionomycin treatment did not induce caspase 3/7 activation. With MERS-CoV infection, significant caspase 3/7 activation was observed at both 24 and 48 h postinfection, but only in MVE (Fig. 3A) and not in FB (Fig. 3B). Caspase activation was not observed with UV-inactivated MERS-CoV treatment or in mock-infected cells for either cell type (Fig. 3A and B). When measuring cell viability within the same assay, neither mock infection nor treatment with UV-inactivated virions appreciably affected cell viability (Fig. 3C and D). Importantly, treatment of both FB and MVE with control compounds (e.g., staurosporine or ionomycin) diminished cell viability, but MERS-CoV infection only diminished cell viability in MVE (Fig. 3C and D). Death of MERS-CoV-infected MVE could lead to permeability at the epithelial/endothelial cell barrier as one of the early steps of ARDS, while viable MERS-CoV-infected FB would continue to produce high levels of infectious virus, both of which would contribute to the overall disease burden in the infected patient.

### Expression of proteins indicative of the UPR pathway is increased in MERS-CoV-infected MVE.

**FIG 4:**
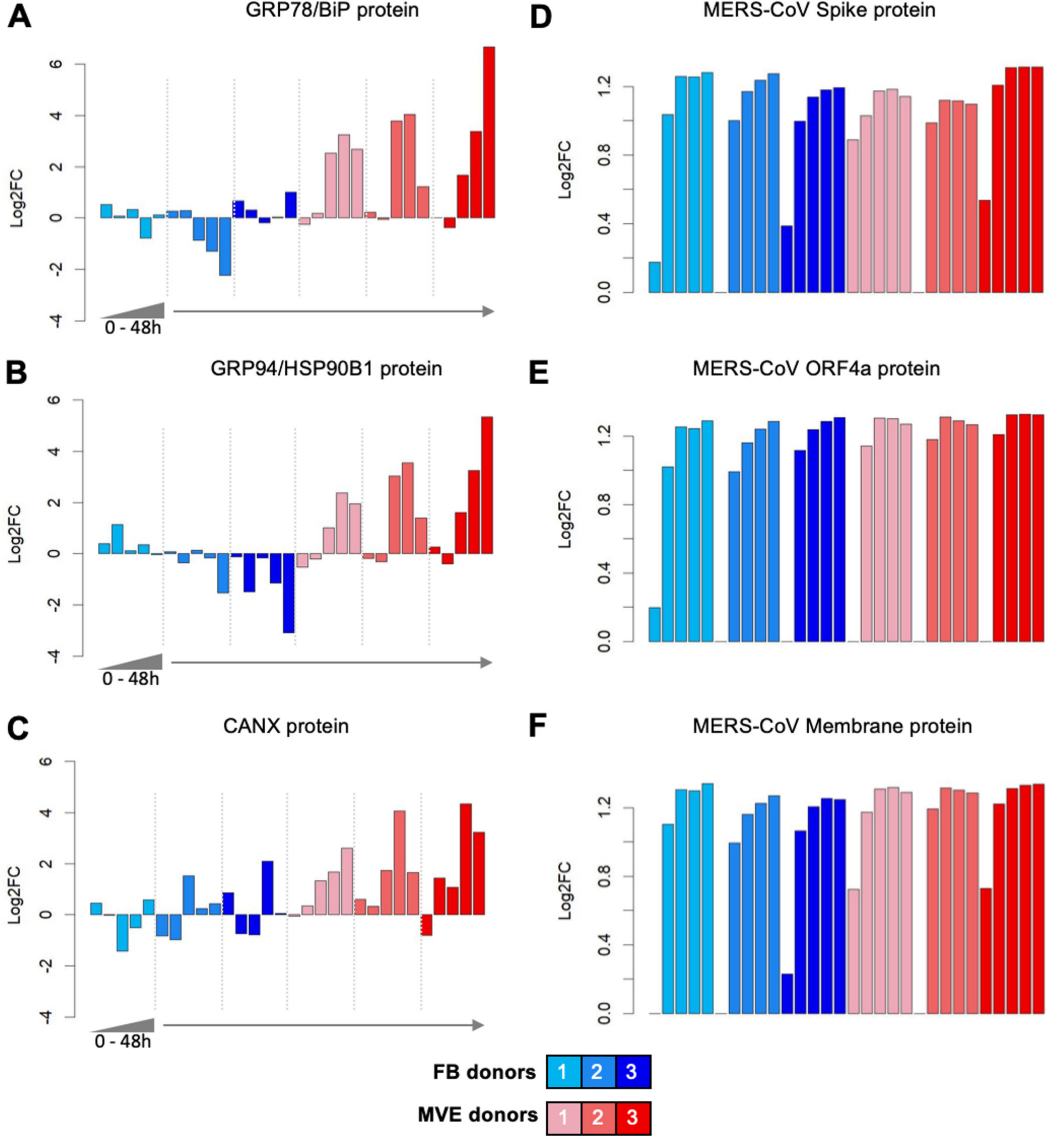
Protein markers suggest activation of unfolded protein response (UPR) in MVE. (A to C) Protein expression of markers of the unfolded protein response. Each figure shows the expression behavior of a UPR marker in FB (blue) and MVE (red), with individual donors represented separately as individual color hues. For each donor, a time course of expression is shown that includes samples taken at 0, 12, 24, 36, and 48 h postinfection. Values are expressed as the log_10_ *P* value of the change between the infection and control conditions, with sign assigned according to the direction of fold change. (D to F) Viral protein expression. MERS-CoV spike, open reading frame 4a (ORF4a), and membrane protein abundances are represented as the ratio of each protein to the average abundance of all other proteins detected in each sample. GRP78, glucose-regulated protein 78; BiP, binding immunoglobulin protein; GRP94, glucose-regulated protein 94; HSP90B1, heat shock protein 90-kDa beta member 1; CANX, calnexin.

Both omics data and confirmatory experimental validation studies suggested that MERS-CoV-infected MVE were dying via apoptotic pathways. To better understand the initiation of this process, we reanalyzed our omics data focusing on specific mediators of apoptosis and UPR. We found increased protein expression of three well-established UPR markers, glucose-regulated protein 78 (GRP78; also known as binding immunoglobulin protein [BiP]), heat shock protein 90-kDa beta member 1 (HSP90B1; also known as GRP94), and calnexin (CANX) in MERS-CoV-infected MVE but not infected FB (Fig. 4A to C). GRP78/BiP is a master regulatory protein for the UPR that binds to regulatory enzymes within the UPR pathway, keeping them inactive unless misfolded proteins accumulate triggering dissociation and enzyme activation. HSP90B1/GRP94 and CANX are ER protein chaperones that facilitate nascent protein folding. In support of the viral titer data, the expression of MERS-CoV structural proteins, spike glycoprotein, and membrane protein as well as the accessory open reading frame protein 4a (ORF4a) were expressed to similar levels in both cell types for all donors by 12 h postinfection (Fig. 4D to F). Cumulatively, these data suggest that MERS-CoV modulation of the UPR is specific to a particular cellular environment (MVE) and may not be broadly applicable to all infected cells and tissues.

### MERS-CoV infection activates stress response pathways in primary human lung MVE and FB.

**FIG 5:**
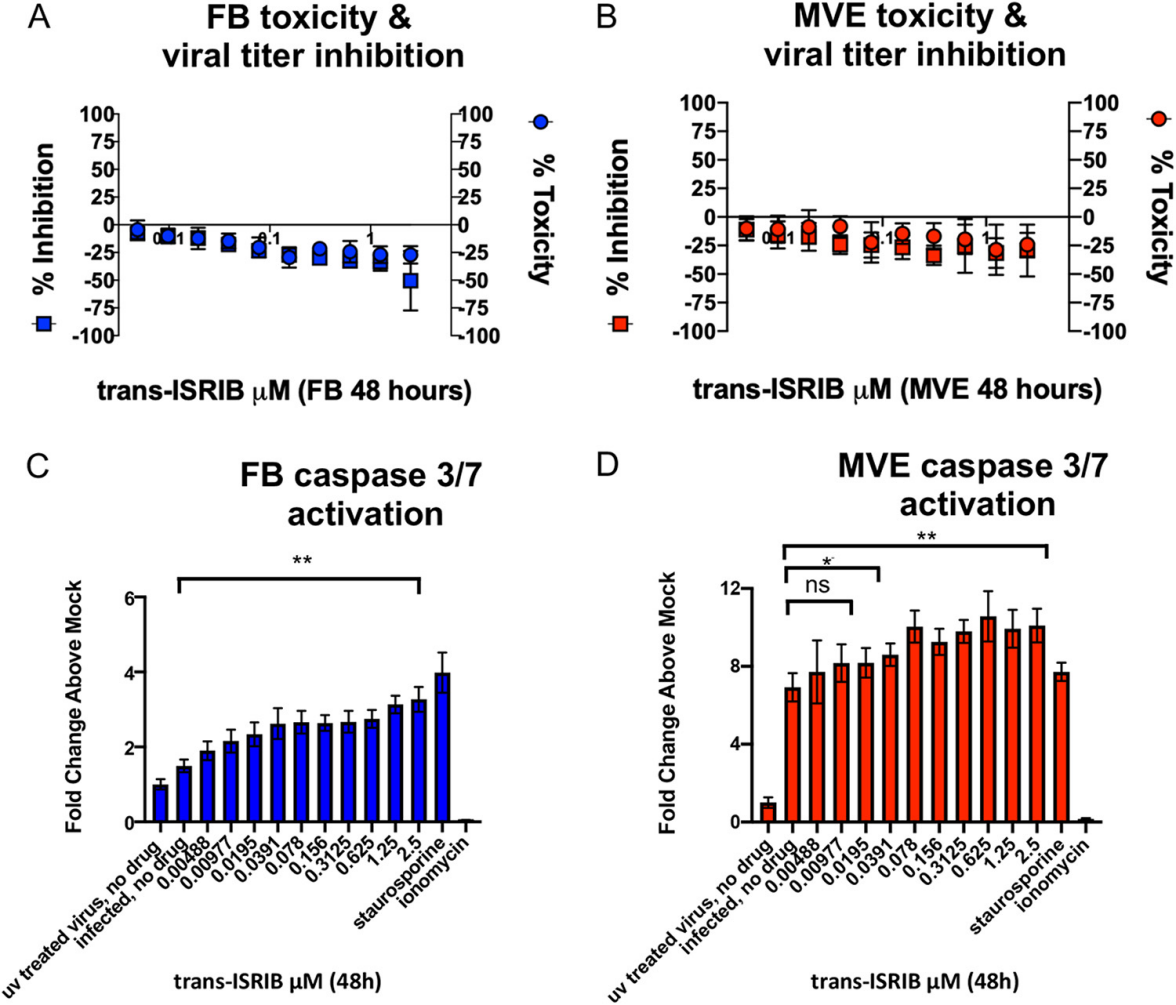
Increased caspase 3/7 activation following treatment with trans-ISRIB and MERS-CoV infection of MVE and FB support the activation of the integrated stress response. (A and B) Donor-matched uninfected FB (circles, A) and MVE (circles, B) were treated with serial dilutions of trans-ISRIB inhibitor (2.5 μM to 0.00488 μM) and cell viability assessed at 48 h posttreatment using Promega’s CellTiter Glo kit according to the manufacturer’s instructions. Each circle represents mean values from two experiments graphed as percent toxicity. Error bars indicate standard deviations from the means. In parallel, the same donor-matched FB (squares, A) and MVE (squares, B) were infected with MERS-nanoluc and simultaneously treated with the same serial dilutions of trans-ISRIB. Nanoluciferase expression was assayed at 48 h postinfection (squares, A and B) using Promega’s NanoGlo kit according to the manufacturer’s instructions. Control wells were either treated with drug diluent and UV-inactivated virus or infected with MERS-nanoluc (MOI, 5) and treated with only drug diluent. Each square represents mean values from two experiments graphed as percent inhibition. Error bars indicate standard deviations from the means. Blue squares, MERS-nanoluc-infected FB treated with trans-ISRIB serial dilutions; blue circles, uninfected FB treated with trans-ISRIB serial dilutions; red squares, MERS-nanoluc-infected MVE treated with trans-ISRIB serial dilutions; red circles, uninfected MVE treated with trans-ISRIB serial dilutions; MVE, primary human lung microvascular endothelial cell; FB, primary human lung fibroblast; MERS-nanoluc, MERS-CoV expressing nanoluciferase. (C and D) Donor-matched FB (C) and MVE (D) were plated and infected with UV-inactivated or wild-type MERS-CoV, and at 48 h postinfection caspase 3/7 activation was determined according to the manufacturer’s instructions using the Apotox Triplex kit. Control uninfected cells were treated with staurosporine (8 μM MVE/10 μM FB, activates caspase 3/7) or ionomycin (50 μM MVE/60 μM FB, does not activate caspase 3/7). Results are graphed as relative light units, and error bars indicate standard deviations from the means. Statistical analysis was performed in GraphPad and determined by Mann-Whitney U test. Blue bars, MERS-CoV-infected FB treated with the indicated compound; red bars, MERS-CoV-infected MVE treated with the indicated compound. *, *P* < 0.03; **, *P* < 0.004; ns, not statistically significant.

Following external stimuli or pathogen invasion, cells will often halt global cellular translation to attempt to reestablish homeostasis (31). Cell viability and transcriptomic and proteomic data suggested that MERS-CoV-infected MVE but not FB have activated cellular stress response (UPR and apoptosis) pathways. To validate activation of cellular stress response pathways in MERS-CoV-infected MVE and FB, lung cells were simultaneously infected and treated with serial doses of the integrated stress response inhibitor trans-ISRIB (32–34). The integrated stress response (ISR) is mediated by one of four kinases (protein kinase R-like endoplasmic reticulum kinase [PERK], heme-regulated inhibitor [HRI], general control nondepressible 2 [GCN2], and double-stranded RNA dependent protein kinase [PKR]) that can all phosphorylate the key translation mediator, elongation initiation factor 2alpha (eIF2alpha) (35). Phosphorylated eIF2alpha shuts down global host translation to allow the cell time to recover from a variety of stressful stimuli; however, if recovery is not possible, then apoptotic processes are initiated. The inhibitor renders cells no longer sensitive to eIF2alpha phosphorylation. In cells that are not stressed, trans-ISRIB has no effect, but in cells with activated stress pathways (specifically PERK activated ones), treatment with trans-ISRIB results in decreased cell viability (34). Dose-response studies were performed in mock- and MERS-CoV-infected MVE and FB with trans-ISRIB (2.5 μM to 0.00488 μM) to determine if pharmacologic perturbation of the ISR would affect MERS-CoV replication and/or cell death (36). We utilized a MERS-CoV nanoluciferase reporter virus (MERS nanoluc) to increase accuracy and throughput of the assay (37). Inhibition of the ISR did not alter MERS-CoV replication in either infected MVE or FB (Fig. 5A and B). In addition to monitoring changes in virus replication in the context of inhibition of the ISR, we also monitored cytotoxicity in drug-treated uninfected cultures via CellTiter-Glo cell viability assay. Treatment with trans-ISRIB was not cytotoxic at any dose tested (Fig. 5A and B). To determine if inhibition of the ISR modifies induction of apoptosis following MERS-CoV infection, we performed dose-response assays similar to those described for Fig. 5A and B but then assayed for caspase 3/7 activation as in Fig. 3 using similar UV-inactivated and small-molecule controls (i.e., staurosporine and ionomycin). Significant dose-responsive changes in caspase 3/7 activation above untreated infected cells were detected for both primary human lung MVE and FB following treatment with trans-ISRIB, suggesting that both cell types activate and benefit from stress response pathways following MERS-CoV infection.

### PERK activation is critical for MERS-CoV replication in primary human lung MVE and FB.

**FIG 6:**
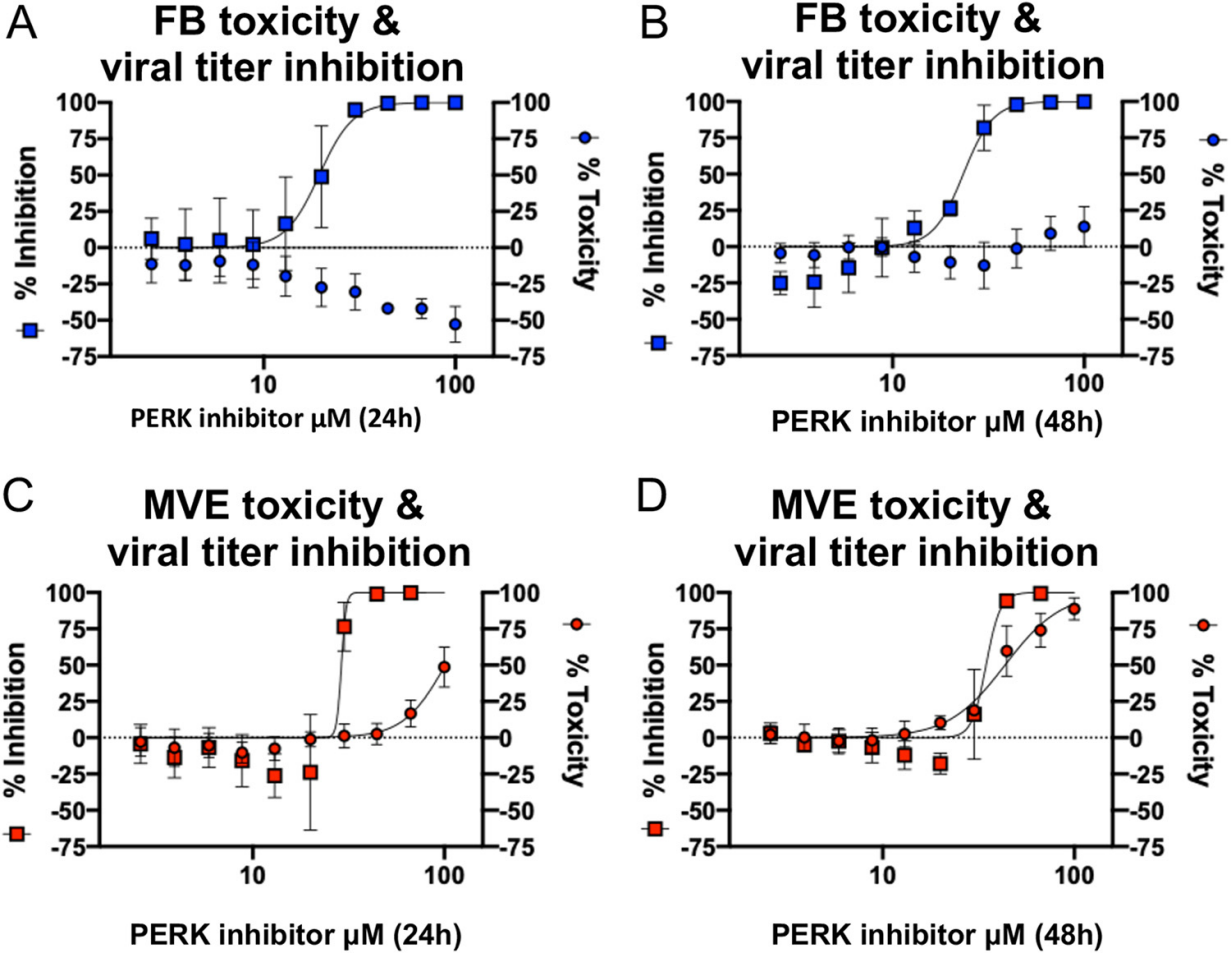
PERK inhibitor AMG PERK 44 reduces MERS-CoV replication in infected primary human lung MVE and FB. (A to D) Donor-matched uninfected FB (circles, A and B) and MVE (circles, C and D) were treated with serial dilutions of PERK inhibitor (100 μM to 2 μM), and cell viability was assessed at 24 (A and C) and 48 (B and D) h posttreatment using Promega’s CellTiter Glo kit according to the manufacturer’s instructions. Each circle represents mean values from two experiments graphed as percent toxicity. Error bars indicate standard deviations from the means. In parallel, the same donor-matched FB (squares, A and B) and MVE (squares, C and D) were infected with MERS-nanoluc and simultaneously treated with the same serial dilutions of PERK inhibitor. Nanoluciferase expression was assayed at 24 (squares, A and C) and 48 (squares, B and D) h postinfection using Promega’s NanoGlo kit according to the manufacturer’s instructions. Control wells were either treated with drug diluent and UV-inactivated virus or infected with MERS-nanoluc (MOI, 5) and treated with only drug diluent. Each square represents mean values from two experiments and are graphed as percent inhibition. Error bars indicate standard deviations from the means. Blue squares, MERS-nanoluc-infected FB treated with PERK inhibitor serial dilutions; blue circles, uninfected FB treated with PERK inhibitor serial dilutions; red squares, MERS-nanoluc infected MVE treated with PERK inhibitor serial dilutions; red circles, uninfected MVE treated with PERK inhibitor serial dilutions. PERK, protein kinase R-like ER kinase; MVE, primary human lung microvascular endothelial cell; FB, primary human lung fibroblast; MERS-nanoluc, MERS-CoV expressing nanoluciferase.

To further validate the results thus far, which suggested that apoptosis of MVE was associated with the UPR, we pharmacologically perturbed the UPR in MERS-CoV-infected MVE and FB using an inhibitor specific to the first enzyme activated following misfolded protein accumulation, PERK (AMG PERK 44). Activated PERK regulates phosphorylation of eIF2alpha in the UPR. Following activation of the UPR and PERK, host translational shutoff serves to reestablish ER homeostasis and proper protein folding, which, if not achieved, results in the activation of apoptotic pathways. Dose-response studies were performed in mock- and MERS-CoV-infected MVE and FB with high doses of PERK inhibitor (AMG PERK 44 at 100 to 2 μM) to determine if pharmacologic perturbation of the PERK arm of the UPR would affect MERS-CoV replication and/or cell death (36). Inhibition of PERK diminished MERS-CoV replication in both FB (Fig. 6A and B) and MVE (Fig. 6C and D) in a dose-dependent manner when assayed at either 24 (Fig. 6A and C) or 48 (Fig. 6B and D) h postinfection using the same MERS-CoV nanoluc assays as those described for trans-ISRIB. In addition to monitoring changes in virus replication in the context of PERK inhibition, we also monitored cytotoxicity in drug-treated uninfected cultures via CellTiter-Glo cell viability assay. Unlike FB, in which we did not observe cytotoxicity at either time point (Fig. 6A and B), in MVE we observed a dose-dependent increase in cytotoxicity with PERK inhibition that was most notable at 48 hpi (Fig. 6D). Importantly, we found that at 24 h postinfection, MERS-CoV replication was inhibited at multiple drug doses that did not exhibit cytotoxicity. These results suggest that primary human lung MVE are more sensitive to inhibition of the UPR in the absence of infection, in contrast to the more tolerant primary lung FB. To determine if PERK inhibition modifies induction of apoptosis following MERS-CoV infection, we performed dose-response assays similar to those described for Fig. 6A to D but then assayed for caspase 3/7 activation as in Fig. 3 using similar UV-inactivated and small-molecule controls (i.e., staurosporine and ionomycin). No significant dose-responsive change in caspase 3/7 activation above untreated infected cells was detected in either primary human lung MVE or FB following MERS-CoV infection and treatment with PERK inhibitor (Fig. S4). Taken together, the results from trans-ISRIB and AMG PERK 44 treatment suggest that both FB and MVE undergo stress response activation during infection, which is exacerbated when the ability to slow translation through eIF2alpha phosphorylation is removed, and that viral production is dependent on non-eIF2alpha-related signaling through PERK kinase.

10.1128/mBio.01572-21.4 FIG S4 Inhibition of PERK by AMG PERK 44 does not alter caspase 3/7 activation following MERS-CoV infection. (A to D) Donor-matched FB (A and B) and MVE (C and D) were plated and infected with UV-inactivated or wild-type MERS-CoV, and at 24 (A and C) and 48 (B and D) h postinfection caspase 3/7 activation was determined according to manufacturer’s instructions using the Apotox Triplex kit. Control uninfected cells were treated with staurosporine (8 μM MVE/10 μM FB, activates caspase 3/7) or ionomycin (50 μM MVE/60 μM FB, does not activate caspase 3/7). Results are graphed as relative light units, and error bars indicate standard deviation from the mean. Statistical analysis was performed in GraphPad determined by Mann-Whitney U test. Download FIG S4, PDF file, 0.1 MB. Copyright © 2021 Sims et al. 2021 Sims et al. https://creativecommons.org/licenses/by/4.0/ This content is distributed under the terms of the Creative Commons Attribution 4.0 International license.

### PERK inhibition improves pulmonary function and diminishes MERS-CoV pathogenesis and ALI.

**FIG 7:**
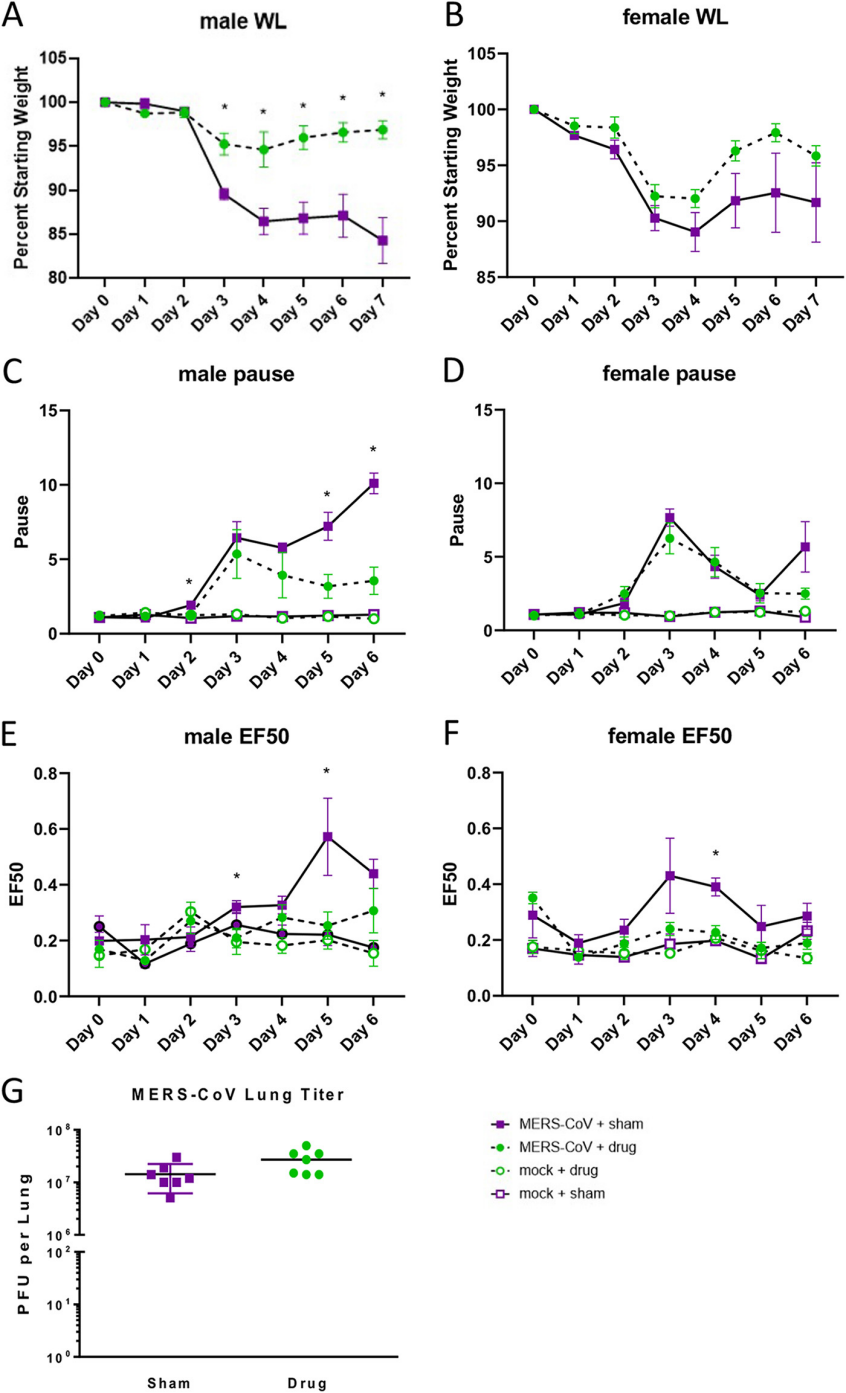
PERK inhibitor AMG PERK 44 treatment decreases MERS-CoV pathogenesis. (A to G) hDPP4 mice were treated with PERK inhibitor AMG PERK 44 or sham control and infected with 5 × 10^4^ PFU of MERS-CoV MAm35c4 or PBS control. (A and B) Weight loss was observed over the course of a 7-day infection. (C to F) Daily respiratory function measurements were taken via whole-body plethysmography. (G) Viral titers were scored at the time of harvest. Error bars indicate standard errors of the means. *, *P*  < 0.05.

**FIG 8:**
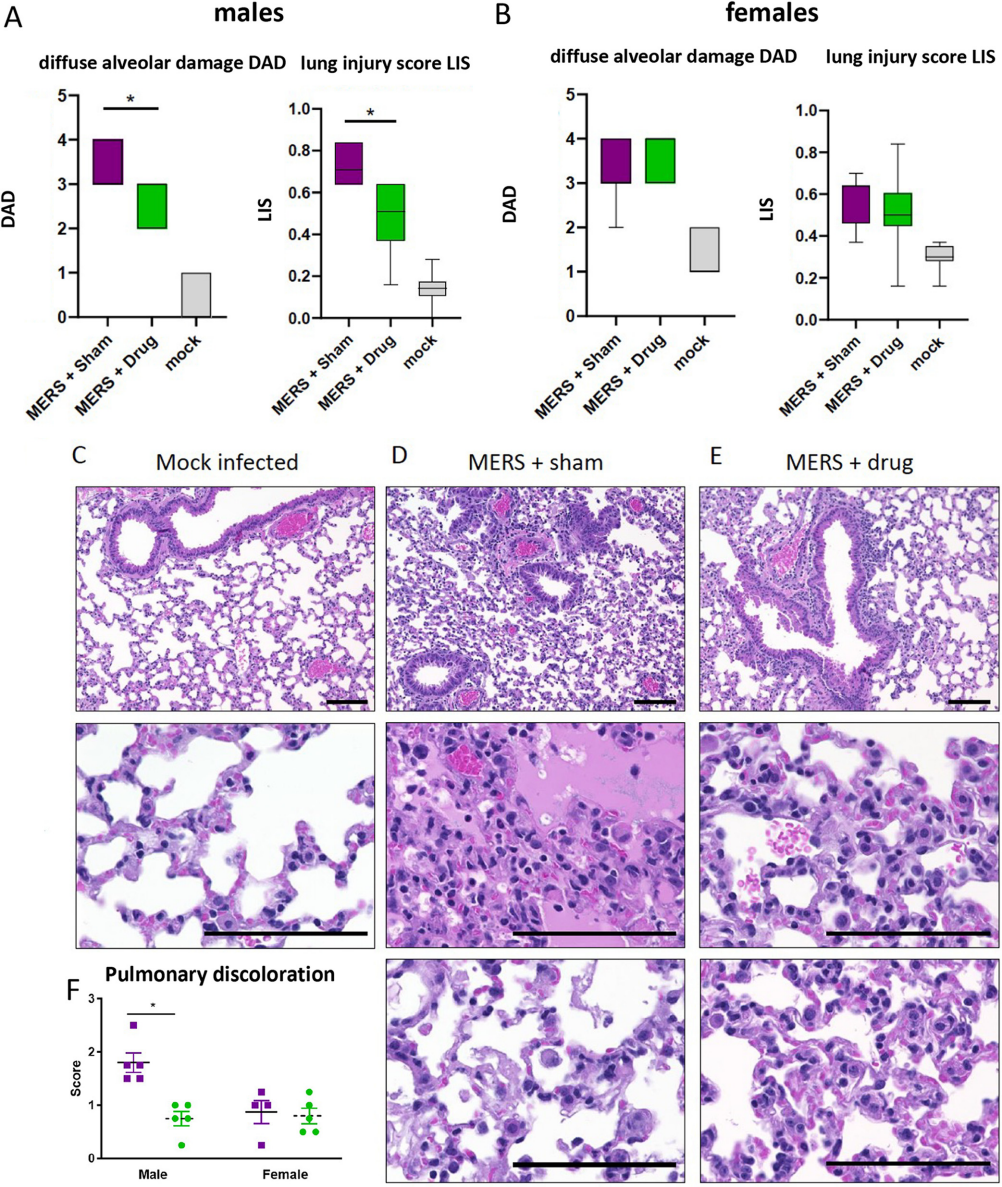
AMG PERK 44 reduces clinical signs of acute lung injury in MERS-CoV challenge model. (A to E) The histological features of acute lung injury were blindly scored using the American Thoracic Society Lung Injury Scoring system by Matute-Bello, creating an aggregate score for neutrophils in the alveolar and interstitial space, hyaline membranes, proteinaceous debris filling the air spaces, and alveolar septal thickening. (A and B) Scoring for ALI and diffuse alveolar damage in male and female mice, respectively. (C to E) Representative lung pathology for mock-infected (C), MERS-CoV-infected and sham-treated (D), and MERS-CoV-infected and AMG PERK 44-treated (E) male mice at 7 days postinfection. (F) Pulmonary discoloration was scored at the time of harvest. *, *P* < 0.001.

Because treatment of primary human lung cells with PERK inhibitor AMG PERK 44 resulted in a significant decrease in MERS-CoV replication in both cell types, we sought to determine if *in vivo* inhibition would result in a change in viral replication and/or pathogenesis. To address this, we utilized a transgenic mouse model where the murine ortholog of the human MERS-CoV receptor, dipeptidyl peptidase 4 (DPP4), was humanized at residues 288 and 330 (hDPP4), facilitating high-titer virus replication localized to the respiratory tract and lung pathology similar to that observed in humans (38, 39). We treated hDPP4 male and female mice with AMG PERK 44 or vehicle at 24 h prior to and after MERS-CoV infection. By 3 days post-MERS-CoV infection, vehicle-treated mice lost more weight than AMG PERK 44-treated mice. This change was apparent in both male and female animals and reached statistical significance in males (Fig. 7A and B). Importantly, AMG PERK 44-treated mice also displayed an improved time to recovery over the study time course (Fig. 7A and B). To determine if PERK inhibition through AMG PERK 44 treatment would affect pulmonary function, we performed whole-body plethysmography (WBP) daily. Concordant with weight loss, AMG PERK 44 treatment improved pulmonary function compared to those receiving vehicle. For three WPB metrics typically induced following MERS-CoV infection (38–40), Pause (Fig. 7C and D), EF50 (Fig. 7E and F), and Penh (Fig. S5A and B), AMG PERK 44-treated mice had improved values over vehicle-treated animals, with time points in both males and females showing significance (*P* < 0.05) among these metrics. Pause, EF50, and PenH are all measures of airway constriction or obstruction and indicate that AMG PERK 44 treatment is relieving or preventing lung pathology that negatively affects lung function. Interestingly, virus titer in the lung on day 3 postinfection was similar regardless of treatment (Fig. 7G) (38–40). To quantitatively assess the impact of AMG PERK 44 treatment on MERS-CoV-infected mice, histological lung sections were assessed using the American Thoracic Society (ATS) scoring system, designed to more closely relate data from small-animal models of acute lung injury (ALI) to infected patient outcomes, as previously described (41, 42). Hematoxylin- and eosin-stained fields of lung tissue were blindly scored for the presence of neutrophils in the alveolar and interstitial space, hyaline membranes, proteinaceous debris filling the airway spaces, and thickening of the alveolar septa (41). ATS lung injury scores were significantly reduced in male but not female MERS-CoV-infected AMG PERK 44-treated mice (Fig. 8A to E). In addition, pulmonary discoloration score, a gross pathological phenotype that increases coincident with MERS-CoV pathogenesis, was significantly diminished in AMG PERK 44-treated male mice compared to those receiving vehicle treatment (Fig. 8F) but not in females. Together with the wide variety of supportive data described above, the *in vivo* studies with AMG PERK 44 directly demonstrate the importance of PERK signaling in promoting MERS-CoV pathogenesis in mice. Our unbiased systems biology-based approach identified a pathway differentially modulated during MERS-CoV infection of primary human lung cells, validated the importance of this pathway for MERS-CoV replication and cell death *in vitro*, and demonstrated its importance in regulating viral pathogenic outcomes *in vivo*, leading to a targeted host-based antiviral therapy.

10.1128/mBio.01572-21.5 FIG S5 PERK inhibitor AMG PERK 44 treatment decreases MERS-CoV pathogenesis. (A and B) hDPP4 mice were treated with PERK AMG PERK 44 or sham control and infected with 5 × 10^4^ PFU of MERS-CoV MAm35c4 or PBS control. Daily respiratory function measurements (PenH) were taken via whole-body plethysmography. Error bars indicate standard error of the mean. * *P* < 0.05. Download FIG S5, PDF file, 0.08 MB. Copyright © 2021 Sims et al. 2021 Sims et al. https://creativecommons.org/licenses/by/4.0/ This content is distributed under the terms of the Creative Commons Attribution 4.0 International license.

## DISCUSSION

Viral pathogenesis is a multifactorial process driven by virus and host factors, responses in infected and neighboring cells, innate and adaptive immunity, host and viral genetics, and responses at the cellular, tissue, organ, and organismal levels. Due to this inherent complexity, defining the relative contribution of each of these factors to the disease state and the underlying cellular mechanisms that contribute to the progression toward severe disease is complicated. Thus, new models are needed to define cell- and tissue-specific host response patterns that play critical roles in viral pathogenesis and for identifying host-based antiviral therapies for disease amelioration. SARS-CoV, SARS-CoV2, and MERS-CoV all cause severe respiratory disease, with replication and pathogenesis primarily limited to the airways and lung. As shown in a recent pathological evaluation of human tissue from a fatal case of MERS-CoV, diffuse alveolar damage, the pathological hallmark of ALI, was noted along with viral antigen primarily found in alveolar pneumocytes with no extrapulmonary viral antigen detected (4). Similar findings have been reported following severe SARS-CoV and SARS-CoV2 infections, suggesting common mechanisms of emerging coronavirus-induced ALI (14, 43). While the few postmortem analyses of tissue samples from MERS-CoV patients have provided insight into the types of cells targeted by virus and the degree and severity of pathology in the lung, many basic questions related to the kinetics of virus replication, cell targeting, and the host response remain unknown. Primary cell cultures offer an opportunity to decrease the complexity of the intact organ to study how virus-host interactions determine the fate of critical cell types following infection. While most studies with pneumotropic viruses have focused on airway epithelial cells or type II pneumocytes, MVE and FB play critical roles in maintaining lung architecture, compartmentalization, and function (44). FB play a central role in the homeostasis of the extracellular matrix and are effector cells during injury repair, while the microvascular endothelium regulates vascular homeostasis, as vascular leakage can result in inefficient gas exchange in the lungs and lead to hypoxia (45). In addition to MERS-CoV, H5N1 and other highly pathogenic influenza viruses also replicate efficiently in endothelial cells, providing diverse model systems to interrogate tissue-specific virus-host interactions and identify novel tissue-specific response patterns that can be targeted to attenuate virus replication and/or disease (24, 46, 47). Understanding the fate of these cells after infection provides a unique opportunity to understand how MERS-CoV-induced disease phenotypes manifest despite limited clinical sample availability. Death of MERS-CoV-infected MVE and epithelial cells (4, 16) could result in the breakdown of the epithelial-endothelial cell barrier and allow vascular leakage of fluids into the lung, leading to alveolar flooding, a hallmark feature of ALI and ARDS often seen in MERS-CoV, SARS-CoV, and SARS-CoV2 patients and mouse models of human disease (11, 38, 48, 49). These findings support a more thorough examination of MERS-CoV lung cell tropism as clinical samples become available. The current findings also highlight that even within the same organ, cell types can respond to infection with very different outcomes and emphasize the value of studying primary cell types in culture.

During viral infection, unfolded and misfolded host and viral proteins accumulate in the endoplasmic reticulum (ER), disrupting ER homeostasis. ER stress is sensed by several ER chaperone proteins (GRP78, etc.), allowing the cells to activate prosurvival responses that are mediated in part by UPR signaling through PERK, the transmembrane protein inositol‐requiring protein‐1 (IRE1α), and the activating transcription factor‐6 (ATF6) (50, 51). If the UPR fails to restore ER homoeostasis, it promotes cell death through the induction of the specific proapoptotic genes, like CHOP and various caspases (52). To date, the impact of highly pathogenic human CoV infection on different lung cell types has been poorly studied in primary human lung cells. Systems biology analysis of omics data sets provides an unbiased approach to define host pathways that are crucial for virus replication and cell fate outcomes following infection, providing novel insights into different tissue- and cell-specific response patterns to viral infection. Functional enrichment analysis of transcriptomic, proteomic, and lipidomic data sets suggested that activation of the UPR promoted apoptotic cell death in MERS-CoV-infected MVE compared with FB cells that displayed a minimal UPR. Analysis of a single data set would have hinted at either activated stress response or specific cell death pathways in MERS-CoV-infected MVE. However, concordant findings across transcriptomic, proteomic, and lipidomic data sets in matched cell types across multiple patient donors suggested key indicators of activated UPR and apoptotic pathways in MERS-CoV-infected MVE cultures, supporting the hypothesis that prolonged UPR signaling led to ER stress, PERK activation, and programmed apoptotic cell death. In addition, individual data streams can provide unique perspectives not seen in other omics platforms. We saw an example of this in the striking activation of the immune response genes in HAE alone, which was only evident in transcriptomics, as well as ceramide and triglyceride increases, which were only observed from lipidomic studies. MERS-CoV infection activates the integrated stress response in both cell types and results in equivalent viral titers and protein expression; however, the robust activation of the UPR and apoptotic responses in MVE, but not in FB derived from the same individual, is puzzling. UPR is activated when the influx of nascent, unfolded polypeptides exceeds the folding capacity of the ER, which may differ between MVE and FB cells, yet viral structural and accessory protein levels were similar in both cell types. We hypothesize that lung FB can maintain ER homeostasis longer than infected MVE due to higher endogenous levels of ER folding chaperone proteins or increased overall ER volume, allowing continued cell viability after MERS-CoV infection. Future studies will probe how primary lung FB survive MERS-CoV infection. Importantly, ceramide and triglyceride are also potent stimulators of ER stress and apoptosis, and these lipids are also highly upregulated following MERS-CoV infection of MVE but much less so in FB or HAE cultures. Increased expression of multiple triglyceride and ceramide species (products of sphingomyelinase pathways) (53, 54) is strongly correlative with virus-induced CPE in mouse models, demonstrating the *in vivo* relevance of these markers as well (55). Future studies will need to focus on evaluating the cell type-specific UPRs to various biochemical stimuli, after contemporary coronavirus and other respiratory virus infections, and identifying which ceramides and triglycerides regulate the UPR and proapoptotic response in primary lung MVE and FB.

Identifying robust host targets for virus infection control is complicated by the array of cell types and functions in different organs, coupled with the different host response patterns that emerge after virus infection. AMG PERK 44 is a selective and potent PERK inhibitor, and treatment at high doses efficiently blocked MERS-CoV replication in FB and MVE cells *in vitro* (36). These studies validate the importance of PERK activation and the UPR as being critical for MERS-CoV replication fitness early in infection, especially in MVE cells. The most likely mechanism for AMG PERK 44 inhibition of MERS-CoV growth is that the PERK signaling pathway and the UPR promote increased membrane reorganization and suppressed host translation that optimizes an environmental milieu for improved virus replication, maturation, and egress. AMG PERK 44 would promote ER stress and translation of host proteins, potentially disrupting virus replication complexes, budding, and release. However, since trans-ISRIB (which prevents phospho-eIF2alpha from slowing translation) did not affect titer, the effect of AMG PERK 44 that we observed *in vitro* is likely more related to PERK-dependent membrane reorganization. Coronavirus infection is associated with extensive membrane reorganization (56, 57). In fact, coronavirus infection has intrinsic membrane remodeling properties and usurps ER membranes to generate double membrane vesicles (DMVs) and other convoluted membrane structures that likely function as prominent sites of coronavirus replication (56, 58, 59). Moreover, coronavirus egress occurs in the endoplasmic reticulum (ER)/Golgi intermediate compartment where coronavirus glycoproteins can induce ER stress and activate the UPR (17, 59–65). Other coronaviruses respond to the PERK signaling cascade activation in a variety of ways, including increased virus-induced apoptosis (60, 61) or decreased viral titers (62–64). PERK activation was detected following overexpression of both SARS-CoV spike and ORF3a, but the effects on replication were not determined (17, 66). In contrast, the SARS-CoV E glycoprotein protected from UPR stress mediated by the IRE1 pathway (67). The roles of MERS-CoV structural and nonstructural proteins are less certain, although ORF4a has been reported to prevent activation of the stress response pathway via protein kinase R (PKR) antagonism (64). Interestingly, a previous study in Huh7 cells that explored the temporal kinome following MERS-CoV infection did not identify PERK as important for MERS-CoV infection, emphasizing the importance of using the most relevant primary human cell types (68).

AMG PERK 44 prophylactic treatment protected MERS-CoV-infected male mice from clinical disease, as evidenced by reduced weight loss, lung discoloration, and ALI signatures, leading to improved respiratory function and time to recovery. It is noteworthy that AMG PERK 44 treatment did not alter viral titers at day 3 postinfection, perhaps reflecting the differential drug susceptibilities of the multiple cell types (e.g., airway epithelium, alveolar epithelial cells, FB, and MVE) that are infected by MERS-CoV *in vivo*. In mice, PERK activation may not be proviral in all MERS-CoV-susceptible cell types and/or the dosing regimen may not have been equivalent in all infected cell types. These results also suggest that PERK plays a role in a damaging pathogenic process *in vivo* where the individual cell types are part of a larger organ-based physiology and that treatment with the PERK inhibitor actually improves pulmonary function. Specifically, suppression of PERK signaling *in vivo* may inhibit PERK-dependent apoptotic signals in the MVE, which could prevent cell death in this tissue and significantly affect disease outcome. This was not observed in the primary human cell experiments, but since PERK signaling can result in autophagy (cell-preserving) or apoptosis (cell-killing) (69), it is not difficult to imagine a distinct outcome in a whole organism compared to the primary cell infection model. Since SARS-CoV and SARS-CoV2 do not infect primary human lung FB and MVE in infection models, it is difficult to predict if PERK inhibition would have the same beneficial effect in mice infected with these viruses. However, a PERK-dependent apoptotic mechanism could occur in epithelial cells during SARS-CoV and/or SARS-CoV2 infection, making a similar effect of PERK inhibition possible, and will be investigated in future studies.

Currently there are limited approved therapeutics to treat highly pathogenic human coronavirus infections associated with the ongoing MERS-CoV epidemic or the 2019 SARS-CoV2 pandemic, so methods to identify novel viral and host targets are recognized as a high priority by the WHO (70). Despite considerable progress, no therapy is presently available to reverse the widespread vascular leak that is observed during ARDS. Additional treatment studies with AMG PERK 44 in the MERS-CoV challenge model are needed to resolve and expand upon the panel of disease-attenuating drug candidates, including remdesivir and EIDD-2801 (37, 42, 71).

SARS-CoV, SARS-CoV2, and MERS-CoV cause more serious disease in males, so it is interesting that prophylactically treated male, but not female, mice had a significant reduction in hallmark ALI disease phenotypes after MERS-CoV infection (72, 73). A more frequent treatment regimen may improve the outcomes noted in the current study, using AMG PERK 44 in combination with robust antivirals (37, 42, 71), and determining the therapeutic window for AMG PERK 44 efficacy against MERS-CoV and SARS-CoV2 as well as exploring other UPR inhibitors (74) are also critical research priorities for the future. Nevertheless, the comprehensive systems biology approach described here led to identification and validation of tissue-specific and critical host pathways that mediated MVE cell death, leading to potential protein kinase-based therapeutic treatment options that were successful in limiting disease phenotypes in mouse models of MERS-CoV-induced ALI. When coupled with the data presented here, modulating the UPR provides a robust strategy to develop novel therapeutics to control infection-mediated vascular permeability, inflammation, and ALI, especially when linked with potent direct-acting antiviral inhibitors of virus replication *in vivo* (37, 42).

## MATERIALS AND METHODS

### Primary human lung cells and immortalized cells.

Primary human lung cells were isolated from distal lung tissue and processed as described previously (24). The lung cells were obtained under protocol number 03-1396, approved by the University of North Carolina at Chapel Hill Institutional Review Board. Human lung microvascular endothelial cells (MVE) were cultured in Vasculife VEGF-MVE endothelial medium (Lifeline Cell Tech). Human lung fibroblasts (FB) were cultured in DMEM-H basal medium (Corning), 1× penicillin-streptomycin (Sigma), 10% fetal bovine serum (Gemini Bio). Medium fetal bovine serum concentrations were reduced to 4% prior to infection. Vero 81 cells were cultured in Dulbecco’s modified essential medium (DMEM; Gibco) with 10% fetal clone II (HyClone) and 1× antibiotic/antimycotic (Gibco).

### Viruses and viral titration.

Wild-type MERS-CoV (EMC 2012 strain), MERS-CoV expressing nanoluciferase (MERS nanoluc), and MERS-CoV expressing the red fluorescent protein (MERS-RFP) were rescued from infectious clones as previously described (24, 37). Vero C81 cells were used to generate viral stocks and to quantitate viral titers following infection (24, 37).

### Infections to collect transcriptomic, proteomic, and lipidomic samples.

Donor-matched HAE, MVE, and FB from three different human tissue donors were infected and samples harvested for systems biology analysis. For transcriptomic analysis, five replicate wells of HAE, MVE, or FB were either mock infected or infected with wild-type MERS-CoV at a multiplicity of infection (MOI) of 5. At 0, 12, 24, 36, and 48 h postinfection, 100 μl of medium/supernatant/apical wash was collected for viral titration assays, medium was removed, and then 0.5 ml (HAE) or 1 ml (FB/MVE) of TRIzol (Invitrogen/ThermoFisher) was added per well. Total RNA samples were sent to Arraystar for downstream analysis. In parallel, an additional five replicate wells were subjected to the MPLEx protocol (chloroform-methanol, 2:1 mix) precipitation as previously described (75, 76), which results in simultaneous extraction of proteins, metabolites, and lipids with concomitant inactivation of virus. All proteomic and lipidomic samples were desiccated and frozen prior to shipment to Pacific Northwest National Laboratories for downstream analysis.

### Transcriptomic analysis.

Scanned images were analyzed using Agilent Feature Extraction software (v11.0.1.1). The limma package for R (available on Bioconductor) was used to perform background correction, quantile normalization (normalizeBetweenArrays), and summarization (avereps) to derive a single normalized intensity value per probe. Outlier samples were detected using principal component analysis and by visual inspection of heatmaps, and all data were reprocessed after removing outlier samples. All data processing for each of the biological replicates was performed independently of the other.

### Proteomic analysis.

Samples for proteomics analysis were prepared for and analyzed using liquid chromatography-mass spectrometry and the accurate mass and time tag approach, as described previously (16). The RMD-PAV algorithm (77) was used to identify any outlier biological samples and was confirmed via Pearson correlation. Peptides with inadequate data for either qualitative or quantitative statistical tests were also removed from the data set (78). The SPANS algorithm (78) was used to identify the best normalization method for each data set. Peptides were evaluated with analysis of variance (ANOVA) with a Dunnett test correction and a Bonferroni-corrected g-test to compare each virus to the associated mock within each time point. To perform signature-based protein quantification, BP-Quant (79), each peptide was categorized as a vector of length equal to the number of viruses being evaluated. If all comparisons for all time points are 0 for a specific virus, it is considered nonchanging and given a value of 0. If there are more time points with an increase in virus to mock than a decrease, it is categorized as +1, and the contrary, −1, is given for a decrease in virus to mock. BP-Quant was run with a default parameter of 0.9. All proteins were then analyzed using the same methodology as that for the peptides, i.e., ANOVA with a Dunnett test correction and a Bonferroni-corrected g-test to compare each virus to the associated mock within each time point.

### Lipidomic analysis.

Samples for lipidomics analysis were prepared for and analyzed using liquid chromatography-tandem mass spectrometry, as described previously (55). Lipids were identified using the in-house tool LIQUID (80), and their quantitative data were extracted using MZmine 2.0 as previously described (81). The RMD-PAV algorithm (77) was also used to identify any outlier biological samples and was confirmed via Pearson correlation. Lipids with inadequate data for either qualitative or quantitative statistical tests were also removed from the data set (78). Median centering was used for normalization. Lipids were evaluated with a standard two-sample *t* test to compare each infected condition to the associated mock within each time point.

### Functional enrichment.

We identified significantly changed features (transcripts, proteins, and lipids) by using an adjusted *P* value of 0.05 as a threshold. For each experimental condition, perturbed features were divided into one list each of up- and downregulated items. Each list was tested against the Gene Ontology (GO) database of gene sets using the EASE-adjusted one-sided Fisher exact test (82), such that each condition is tested for up- and downregulation of each function/pathway in the ontology database. Results across multiple conditions were visualized in heatmaps using the log_10_ *P* values of the significance tests, colored to indicate the direction of change. For lipids, feature sets were generated by categorizing the lipids observed in our experiments according to broad lipid classifications; these classifications were then used as gene sets, and the enrichment analysis was then performed in the same manner as that for transcripts and proteins.

### Cell viability and percent infection assays.

To assess numbers of viable cells following MERS-CoV infection of FB and MVE, we used the CellTiter-Glo luminescent cell viability assay (Promega) at 24 and 48 h postinfection/treatment according to the manufacturer’s instructions, and numbers of relative light units were determined by Spectromax plate reader (Molecular Devices), averaged, and plotted. Percent infection was determined using MVE and FB infected with MERS-RFP (MOI, 5) or mock infected and counting nuclei (Hoecht stain diluted 1:5,000 in medium) in replicate wells at 0, 12, 24, 36, and 48 h postinfection. Cell numbers were determined using the Fiji (83) cell counter function, and total numbers of cells versus numbers of red fluorescent cells were determined for at least three wells per condition per cell type. Statistical significance was determined by Mann-Whitney U test (performed in GraphPad Prism).

### Apoptosis assays.

Infected FB and MVE (wild-type MERS-CoV, MOI of 5) were assayed using either the Caspase-Glo 3/7 kit (Promega) to measure caspase activation or using the ApoTox-Glo Triplex assay (Promega) to assess cell viability, cytotoxicity, and caspase 3/7 activation in a single set of samples at 24 and 48 h postinfection according to the manufacturer’s instructions. Control wells were treated with UV-inactivated virus stocks, staurosporine (8 μM for MVE or 10 μM for FB; Sigma), or ionomycin (40 μM for MVE and 60 μM for FB; ThermoFisher). Relative light unit numbers or emission excitation wavelengths were determined by a Spectromax plate reader (Molecular Devices). Statistical significance was determined by Mann-Whitney U test (performed in GraphPad Prism).

### Inhibitor studies.

MERS-CoV-infected MVE and FB were tested at 24 and 48 h postinfection following treatment with a dilution series of trans-ISRIB (Tocris [33, 84, 85], 2.5 μM to 0.00488 μM) or PERK inhibitor (Tocris AMG PERK 44 [36, 86], 100 μM to 2 μM) and was evaluated for cytotoxicity with drug treatment alone (cell viability assay, no virus), activation of the death caspases 3/7 (wild-type MERS-CoV, MOI of 5), and viral replication (detection of luciferase as a surrogate for replication, MERS nanoluc virus, MOI of 5). All assay results were read by SpectraMax (Molecular Devices). Results are graphed as percent inhibition and toxicity (effective and cytotoxic concentration 50) or fold change above mock (caspase activation), as determined by GraphPad Prism.

### Mouse studies.

hDPP4 mice (38) were housed and bred in accordance with the University of North Carolina Department of Comparative Medicine, AALAC number 329. For a single group of mice, dosing occurred once a day 24 h prior to infection, and a second single dose was administered 24 h postinfection via the intraperitoneal route. Male and female average weights were 28 and 21 g, respectively, and the dose of 12 mg/kg of body weight of PERK AMG PERK 44 (Tocris) suspended in water or with a sham control (36, 86) was administered accordingly. Immediately prior to infection, mice were anesthetized with 50 μl of ketamine-xylazine mixture via intraperitoneal injection. Sixteen- to 20-week-old mice were intranasally infected with 10^4^ PFU of mouse-adapted MERS-CoV maM35c4 diluted in OPTIMEM (39); a total volume of 50 μl was given. Mice were weighed daily following infection and had their respiratory function measured 1 day prior to infection and at days two through six postinfection using Buxco whole-body plethysmography (Data Sciences International) (40). Briefly, mice were allowed to acclimate for 30 min in the plethysmography chamber located within the biological safety cabinet, after which measurements were taken for 5 min. Readings were collected every 2 s for a total of 150 measurements per mouse per day. At days three and seven postinfection, mice were euthanized via isoflurane overdose, after which lung tissue was harvested to determine viral replication titers. Gross pulmonary discoloration was observed and scored from zero (none) to four (severe and total) at the time of dissection.

### Statistical analysis.

All statistical data analyses were performed in GraphPad Prism 8. Statistical significance for each endpoint was determined with specific statistical tests. For each test, a *P* value of <0.05 was considered significant. Specific tests are noted in each figure legend.

### Ethical use of laboratory animals.

Efficacy studies were performed under biosafety level three containment in a facility approved for the use of rodents at the University of North Carolina at Chapel Hill (UNC). All work was conducted with protocols approved by the UNC Institutional Animal Care and Use Committee (protocol number 17-294) according to guidelines sets by the Association for the Assessment and Accreditation of Laboratory Animal Care and the United States Department of Agriculture.

### Data availability.

All data sets used for these analyses can be found at https://data.pnnl.gov under the following numbers: MERS-CoV-infected fibroblasts, 13095 (FB donor 1), 13096 (FB donor 2), and 13097 (FB donor 3); MERS-CoV-infected microvascular endothelial cells, 13102 (MVE donor 1), 13103 (MVE donor 2), and 13104 (MVE donor 3); MERS-CoV-infected human airway epithelial cell cultures (HAE), 1661938 (HAE donor 1), 1661939 (HAE donor 2), and 1661940 (HAE donor 3).

10.1128/mBio.01572-21.6 TABLE S1 Enrichment results from transcriptomic data represented in Fig. 1. Download Table S1, XLSX file, 0.02 MB. Copyright © 2021 Sims et al. 2021 Sims et al. https://creativecommons.org/licenses/by/4.0/ This content is distributed under the terms of the Creative Commons Attribution 4.0 International license.

10.1128/mBio.01572-21.7 TABLE S2 Enrichment results from proteomic data represented in Fig. 1. Download Table S2, XLSX file, 0.01 MB. Copyright © 2021 Sims et al. 2021 Sims et al. https://creativecommons.org/licenses/by/4.0/ This content is distributed under the terms of the Creative Commons Attribution 4.0 International license.
