# Supplementary material for: Unfolded Protein Response Inhibition Reduces Middle East Respiratory Syndrome Coronavirus-Induced Acute Lung Injury
Source: mBio. 2021 Aug 10;12(4):e01572-21. doi: 10.1128/mBio.01572-21 (PMC8406233; doi:10.1128/mBio.01572-21)

Supplemental Figure 1 Total Cell Counts for MERS-CoV Infected Primary Human Lung MVE and FB

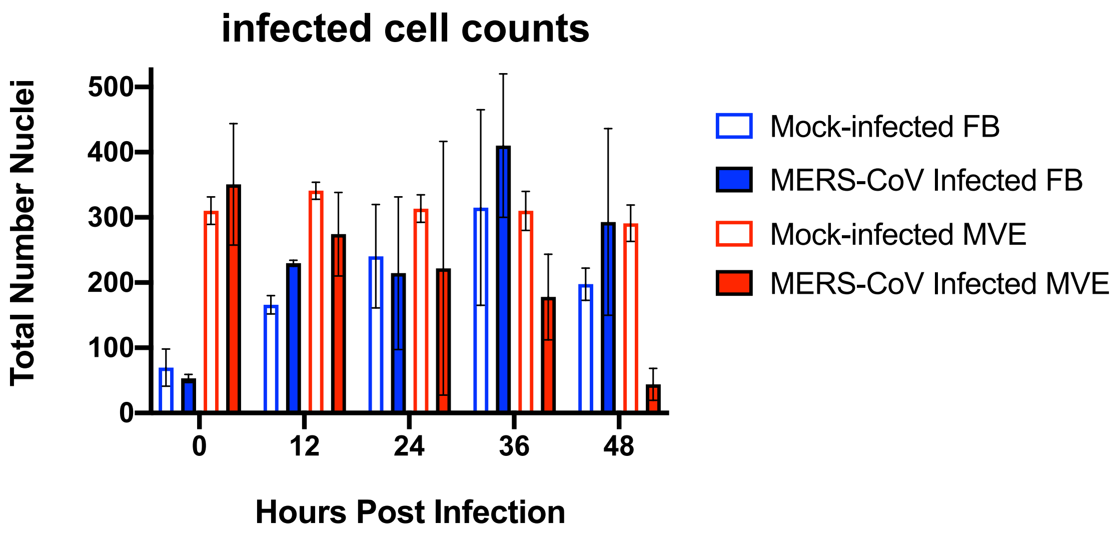

Supplement: FIG S1 [file mbio.01572-21-sf001.pdf]
