# Supplementary material for: Unfolded Protein Response Inhibition Reduces Middle East Respiratory Syndrome Coronavirus-Induced Acute Lung Injury
Source: mBio. 2021 Aug 10;12(4):e01572-21. doi: 10.1128/mBio.01572-21 (PMC8406233; doi:10.1128/mBio.01572-21)
Supplement: FIG S2 [file mbio.01572-21-sf002.pdf]

Supplemental Figure 2 Cell Viability Data with all Donors

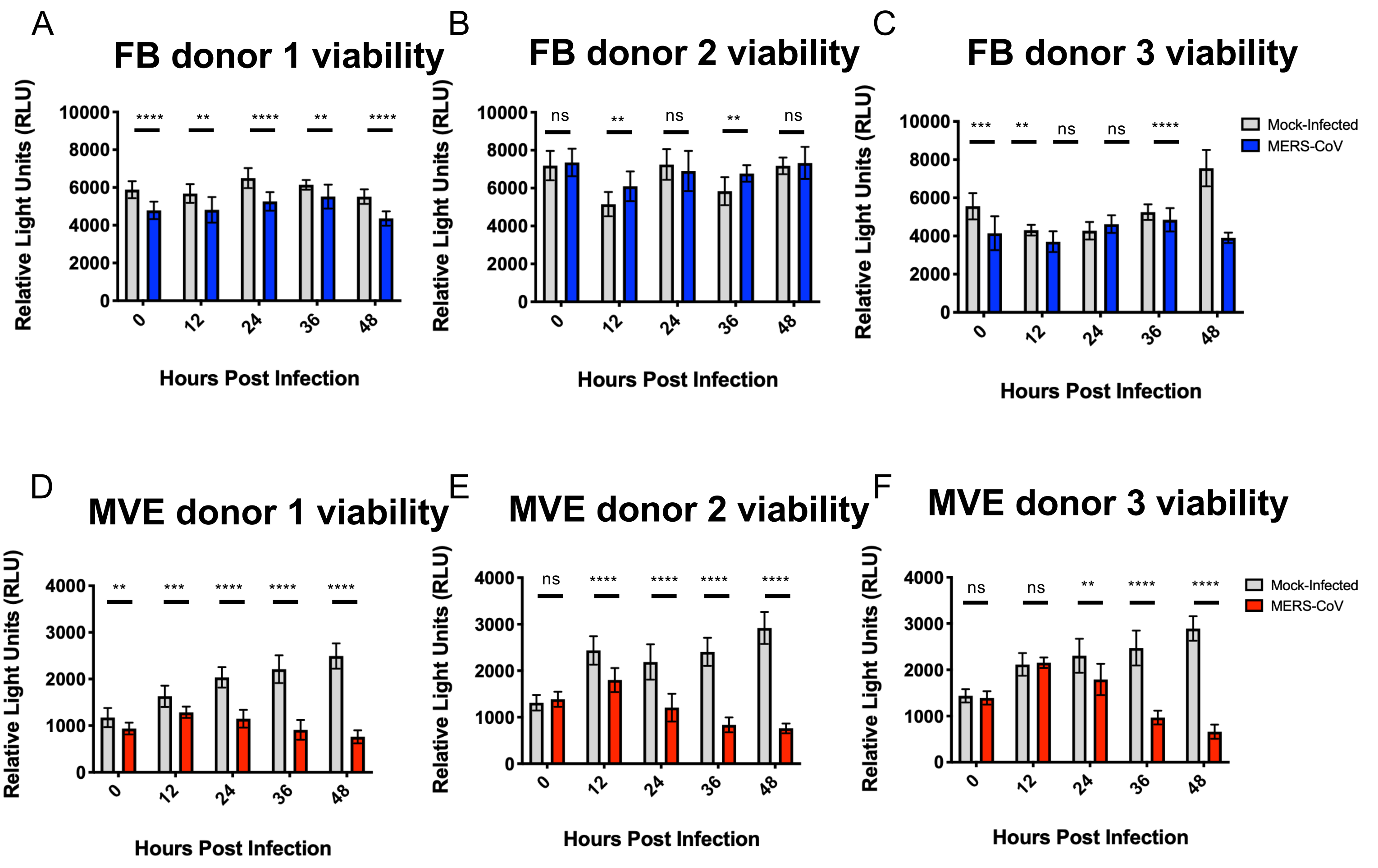

ns not significant
** p= < 0.002
*** p= < 0.0002
****p= < 0.0001

MOI 5
001, 002, 003
individual
tissue donors
