# Supplementary material for: Unfolded Protein Response Inhibition Reduces Middle East Respiratory Syndrome Coronavirus-Induced Acute Lung Injury
Source: mBio. 2021 Aug 10;12(4):e01572-21. doi: 10.1128/mBio.01572-21 (PMC8406233; doi:10.1128/mBio.01572-21)

Supplemental Figure 3 Neither SMAD7 nor FGF2 expression appear to explain cell death phenotype in MERS-CoV infected MVE

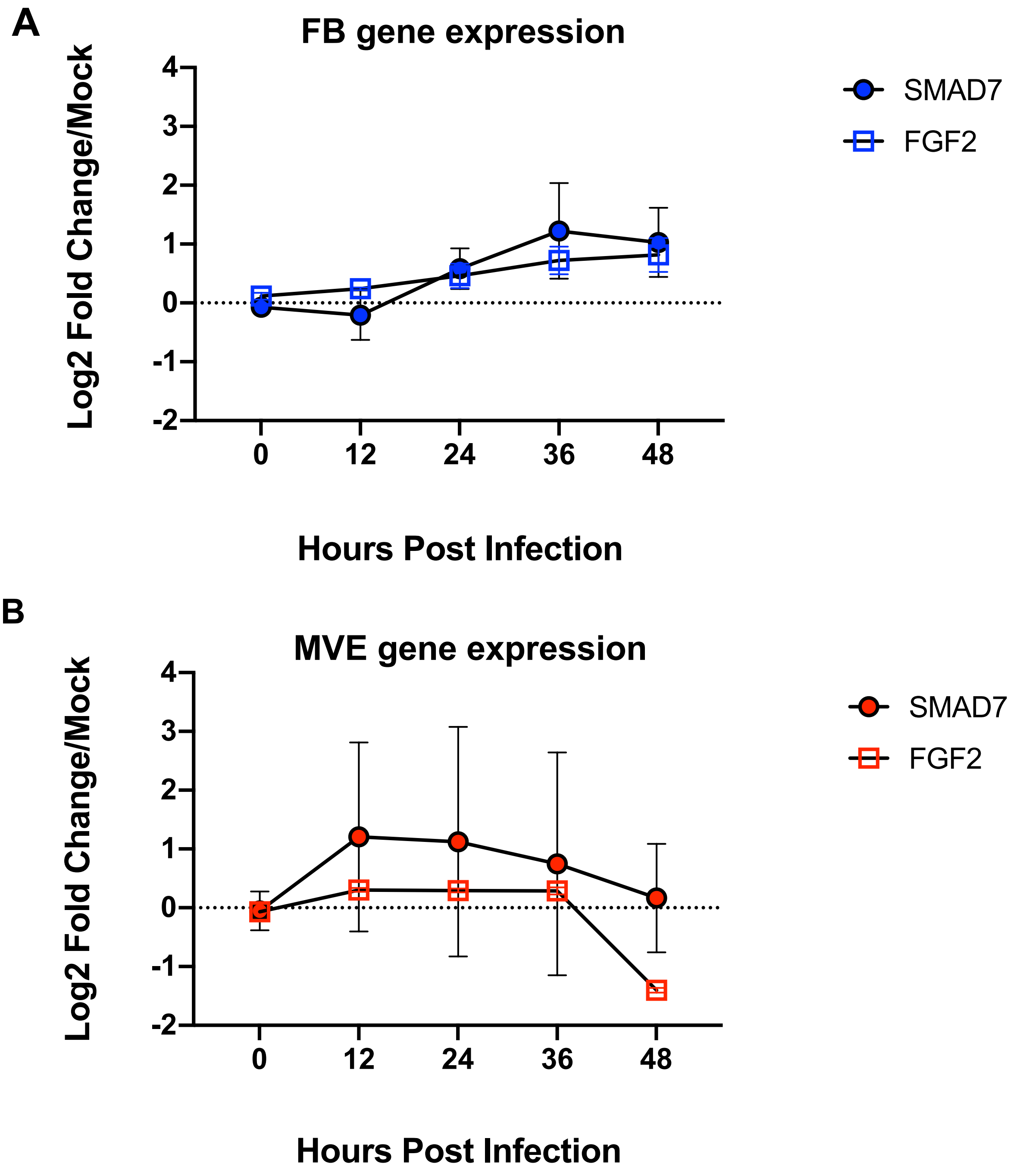

Supplement: FIG S3 [file mbio.01572-21-sf003.pdf]
