# Supplementary material for: Unfolded Protein Response Inhibition Reduces Middle East Respiratory Syndrome Coronavirus-Induced Acute Lung Injury
Source: mBio. 2021 Aug 10;12(4):e01572-21. doi: 10.1128/mBio.01572-21 (PMC8406233; doi:10.1128/mBio.01572-21)
Supplement: FIG S4 [file mbio.01572-21-sf004.pdf]

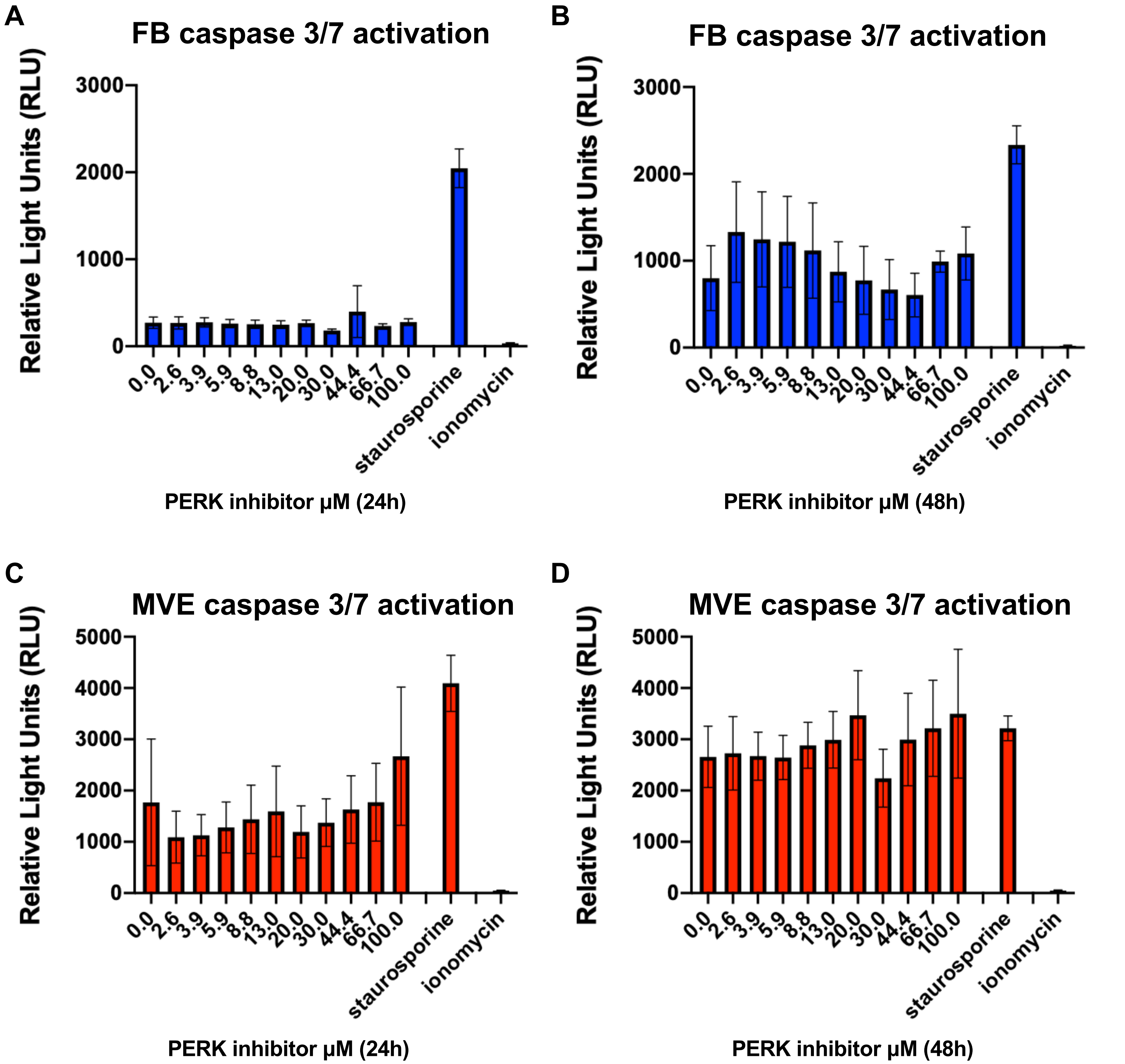

Supplemental Figure 4 Inhibition of PERK by AMG44 does not alter caspase 3/7 activation following MERS-CoV infection.
