# Supplementary figures and images for: Unfolded Protein Response Inhibition Reduces Middle East Respiratory Syndrome Coronavirus-Induced Acute Lung Injury
Source: mBio. 2021 Aug 10;12(4):e01572-21. doi: 10.1128/mBio.01572-21 (PMC8406233; doi:10.1128/mBio.01572-21)

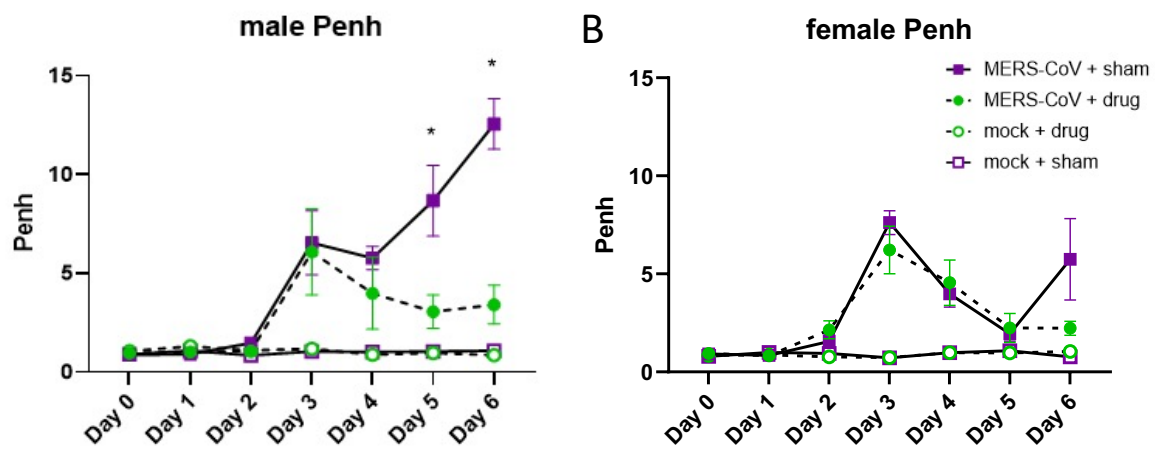

Supplemental Figure 5 Effect of PERK inhibition by AMG44 on Penh in infected mice.

Supplement: FIG S5 [file mbio.01572-21-sf005.pdf]
